# Neonatal mortality risk for vulnerable newborn types in 15 countries using 125.5 million nationwide birth outcome records, 2000–2020

**DOI:** 10.1111/1471-0528.17506

**Published:** 2023-05-08

**Authors:** Lorena Suárez‐Idueta, Hannah Blencowe, Yemisrach B Okwaraji, Judith Yargawa, Ellen Bradley, Adrienne Gordon, Vicki Flenady, Enny S. Paixao, Mauricio L. Barreto, Sarka Lisonkova, Qi Wen, Petr Velebil, Jitka Jírová, Erzsebet Horváth‐Puhó, Henrik Toft Sørensen, Luule Sakkeus, Liili Abuladze, Khalid A. Yunis, Ayah Al Bizri, Arturo Barranco, Lisa Broeders, Aimée E. van Dijk, Fawziya Alyafei, Tawa O. Olukade, Neda Razaz, Jonas Söderling, Lucy K. Smith, Elizabeth S. Draper, Estelle Lowry, Neil Rowland, Rachael Wood, Kirsten Monteath, Isabel Pereyra, Gabriella Pravia, Eric O. Ohuma, Joy E. Lawn, the National Vulnerable Newborn Mortality Collaborative Group and Vulnerable Newborn Measurement Core Group, Kara Warrilow, Harriet Lawford, Arturo Barranco Flores, Jesus Felipe Gonzalez Roldan, Mai AlQubaisi, Tawa O. Olukade, Hamdy A. Ali, Bradley N. Manktelow, Ruth J. Matthews, Alan Fenton, Celina Davis, Bob Black, Joanne Katz, Dan Erchick, Elizabeth Hazel, Mike Diaz, Anne C. C. Lee

**Affiliations:** ^1^ Mexican Society of Public Health Mexico City Mexico; ^2^ Maternal, Adolescent, Reproductive & Child Health (MARCH) Centre London School of Hygiene & Tropical Medicine London UK; ^3^ Faculty of Medicine and Health University of Sydney Sydney New South Wales Australia; ^4^ Centre of Research Excellence in Stillbirth, Mater Research Institute The University of Queensland Brisbane Queensland Australia; ^5^ Centre for Data Integration and Knowledge for Health (CIDACS) Instituto Gonçalo Moniz, Fiocruz Bahia, Fundação Oswaldo Cruz Salvador Brazil; ^6^ Department of Obstetrics & Gynaecology University of British Columbia Vancouver British Columbia Canada; ^7^ Department of Obstetrics and Gynaecology Institute for the Care of Mother and Child Prague Czech Republic; ^8^ Department of Data Analysis Institute of Health Information and Statistics of the Czech Republic Prague Czech Republic; ^9^ Department of Clinical Epidemiology Aarhus University and Aarhus University Hospital Aarhus N Denmark; ^10^ School of Governance, Law and Society, Estonian Institute for Population Studies Tallinn University Tallinn Estonia; ^11^ Finnish Population Research Institute, Väestöliitto Helsinki Finland; ^12^ Department of Paediatrics and Adolescent Medicine American University of Beirut Beirut Lebanon; ^13^ Directorate of Health Information, Ministry of Health Mexico City Mexico; ^14^ Perined Utrecht The Netherlands; ^15^ Hamad Medical Corporation Doha Qatar; ^16^ Clinical Epidemiology Division, Department of Medicine Solna Karolinska Institutet Stockholm Sweden; ^17^ Department of Population Health Sciences, College of Life Sciences University of Leicester Leicester UK; ^18^ School of Natural and Built Environment Queen's University Belfast Belfast UK; ^19^ Queen's Management School Queen's University Belfast Belfast UK; ^20^ Public Health Scotland Edinburgh UK; ^21^ Usher Institute University of Edinburgh Edinburgh UK; ^22^ Pregnancy, Birth and Child Health Team Public Health Scotland Edinburgh UK; ^23^ Department of Wellness and Health Catholic University of Uruguay Montevideo Uruguay

**Keywords:** neonatal mortality, preterm birth, size for gestational age, vulnerable newborn

## Abstract

**Objective:**

To compare neonatal mortality associated with six novel vulnerable newborn types in 125.5 million live births across 15 countries, 2000–2020.

**Design:**

Population‐based, multi‐country study.

**Setting:**

National data systems in 15 middle‐ and high‐income countries.

**Methods:**

We used individual‐level data sets identified for the Vulnerable Newborn Measurement Collaboration. We examined the contribution to neonatal mortality of six newborn types combining gestational age (preterm [PT] versus term [T]) and size‐for‐gestational age (small [SGA], <10th centile, appropriate [AGA], 10th–90th centile or large [LGA], >90th centile) according to INTERGROWTH‐21st newborn standards. Newborn babies with PT or SGA were defined as small and T + LGA was considered as large. We calculated risk ratios (RRs) and population attributable risks (PAR%) for the six newborn types.

**Main outcome measures:**

Mortality of six newborn types.

**Results:**

Of 125.5 million live births analysed, risk ratios were highest among PT + SGA (median 67.2, interquartile range [IQR] 45.6–73.9), PT + AGA (median 34.3, IQR 23.9–37.5) and PT + LGA (median 28.3, IQR 18.4–32.3). At the population level, PT + AGA was the greatest contributor to newborn mortality (median PAR% 53.7, IQR 44.5–54.9). Mortality risk was highest among newborns born before 28 weeks (median RR 279.5, IQR 234.2–388.5) compared with babies born between 37 and 42 completed weeks or with a birthweight less than 1000 g (median RR 282.8, IQR 194.7–342.8) compared with those between 2500 g and 4000 g as a reference group.

**Conclusion:**

Preterm newborn types were the most vulnerable, and associated with the highest mortality, particularly with co‐existence of preterm and SGA. As PT + AGA is more prevalent, it is responsible for the greatest burden of neonatal deaths at population level.

## 1 INTRODUCTION

In 2021, 2.3 million liveborn babies died within the first 28 days of life (neonatal deaths).^1^, ^2^ Worldwide, over 80% of these newborn deaths are in low birthweight (LBW) babies, two‐thirds of which are preterm (<37 weeks).^3^ Defined as less than 2500 g, LBW has been used for more than a century as a marker of vulnerability for newborns, yet the Global Nutrition Plan target for a 30% reduction in LBW is off track.^4^ LBW is the result of being born preterm or small for gestational age (SGA) i.e. below the tenth centile of birthweight for gestational age and sex, or both.^4^ Babies who are born preterm or SGA have an increased risk of complications including neonatal morbidity and mortality, stunting and developmental delay in childhood and long‐term chronic conditions.^5^, ^6^, ^7^, ^8^, ^9^, ^10^ Traditionally, preterm birth and SGA have been described as separate conditions even though they may co‐exist. Each of these classifications alone is not granular enough to understand varying risks for individual small newborns.^11^ For example, newborns born preterm and SGA simultaneously are at particularly high risk of severe clinical complications, requiring neonatal intensive care or leading to death compared with those who are preterm and appropriate for gestational age (AGA, 10th–90th centiles).^12^, ^13^ Whereas the smallest are at the highest risk, it is also important from a public health perspective to understand which groups of babies contribute to the highest levels of mortality at a population level.^14^

In 2020, as part of the *Lancet* Small Vulnerable Newborn Series, a set of newborn types were proposed to advance the classification of newborn vulnerability, by considering gestational age, birthweight and size for gestational age in the same individual.^15^ In addition to the well‐described risk of small babies, being large for gestational age (LGA, >90th centile) has been associated with birth trauma, hypoglycaemia, hospitalisation, overweight and obesity.^16^, ^17^, ^18^ Therefore, categorising each baby based on gestational age (term [T] versus preterm [PT]) and size for gestational age (SGA, AGA and LGA) could enable a more detailed investigation of neonatal vulnerabilities and their potential causal pathways.^19^ This comprehensive identification of newborn types could be useful to implement targeted interventions at the individual clinical and public health levels to improve progress for children, ensuring no one is left behind and all newborns survive and thrive.

This paper aims to fulfil three objectives, namely to quantify the neonatal mortality risk and population attributable risks (PAR%) associated with the following groupings: (1) birthweight categories, (2) gestational age categories and (3) newborn types with six categories combining gestational age (PT versus T) and size for gestational age (SGA, AGA, LGA) in the same individual (Table 1).

**TABLE 1:** Key findings.

**1. What was known?**
Babies born preterm (<37 weeks), Small for gestational age (SGA, <10th centile), and Large for Gestational Age (LGA, >90th centile) are at higher risk of dying during the neonatal period. Previous studies have usually estimated the association of preterm birth, SGA, and LGA with neonatal mortality separately even though these conditions can overlap.
**2. What was done that is new?**
In this study, we used 15 national livebirth and linked neonatal death datasets collected between 2000 to 2020 to compare neonatal mortality and population attributable fractions associated with strata of birthweight, gestational age, and newborn types combining information on gestational age (preterm (PT), or term (T)) and size for gestational age (SGA, appropriate‐for‐gestational age (AGA), LGA). Six newborn types were defined: four small (PT + SGA, PT + AGA, PT + LGA, T + SGA), one large (T + LGA), and one reference (T + AGA).
**3. What was found?**
Our pooled dataset of 125.5 million livebirths from 15 countries provides the first multi‐country mortality estimates of these newborn types. Of the six newborn types, babies born preterm and SGA (PT+SGA) had the highest risk of neonatal death (median relative risk: 67.2, interquartile range, IQR, 45.6, 73.9), but this group are low prevalence. Hence at the population level, most neonatal deaths were attributable to PT + AGA newborn type (median population attributable risk (PAR%): 53.7, IQR 44.5, 54.9). Mortality was highest among babies born <28 weeks and those <1000 g (median risk ratio (RR) ≥ 280‐fold).
**4. What next?**
*Action in preventive programmes*: These six newborn types are relevant for identifying the most vulnerable newborn babies at the clinical level (PT+SGA), and the greatest contributors to neonatal mortality at the population level (PT+AGA). *Research gaps*: Additional analyses of newborn types in lower‐income settings, such as South Asia where SGA rates are very high is needed. Innovative use at the bedside could help target interventions and improve care. Cohort analyses using these types would be valuable to provide more granular information than LBW alone for non‐fatal lifecourse outcomes including non‐communicable conditions.

## 2 METHODS

### 2.1 Compilation of data sets

We aimed to identify population‐based data using routine data of births and neonatal deaths of babies born between 1 January 2000 and 31 December 2020. Potential collaborators and government agencies with national individual‐level data sets with high population‐level coverage (including more than 80% of births in the country) were invited to participate in a new collaboration focused on the multi‐country description of types of vulnerable newborn babies (Vulnerable Newborn Measurement Collaboration). An open call was published in a *Lancet* comment^15^ and widely disseminated through email lists, social media and by contacting authors who had previously published analyses using national routine administrative data sets.

Teams with data sets including live‐birth records and meeting criteria provided analyses to describe the national prevalence of newborn types, as published in another paper on this series.^20^ Among these countries, those with information on neonatal deaths formed a subgroup to perform further analyses on neonatal mortality, which is the focus of this paper.

This is a retrospective analysis of routinely collected data and therefore we followed the Reporting guidelines of studies Conducted using Observational Routinely‐collected Data, the RECORD checklist (Table S1). Ethical approval is summarised in Table S2 for all 15 participating countries and a summary of relevant definitions used is presented in Table S3.

### 2.2 Inclusion and exclusion criteria

**FIGURE 1:**
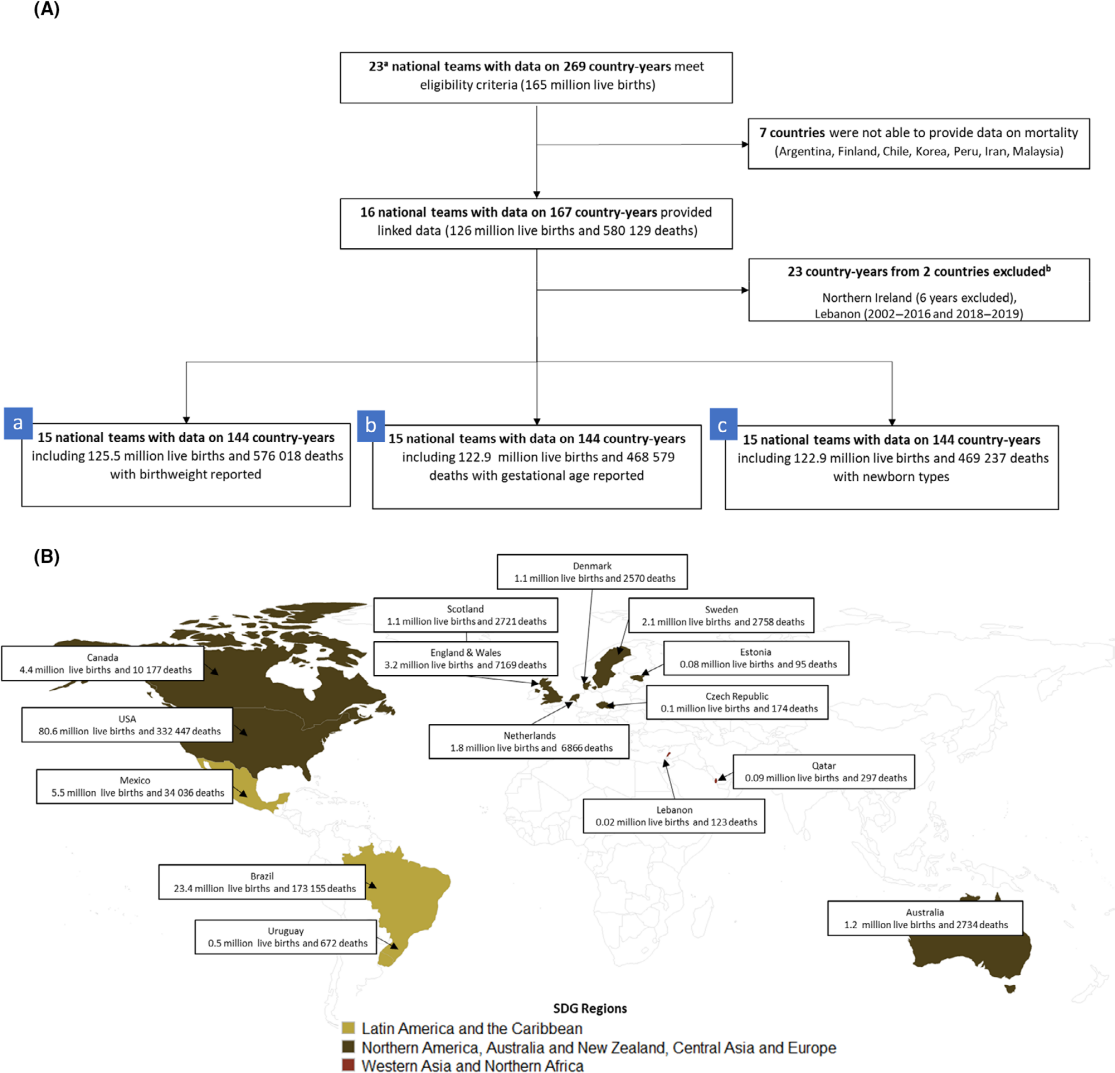
Input data set of Vulnerable Newborn Mortality study. (A) Flowchart. (B) Number of live births in millions and neonatal deaths, by country. ^a^Twenty‐three countries from the Vulnerable Newborn Collaboration were invited to participate in the Mortality study.^20^ ^b^Lebanon 2002–2016 and 2018–2019 were excluded due to the lack of availability of neonatal mortality reported to UNIGME, Northern Ireland was excluded because we were not able to calculate neonatal mortality rate due to small, masked cells. Map legends show the distribution of the 125.5 million babies with birthweight recorded included in these analyses.

We included national data sets compiled for the Vulnerable Newborn Measurement Collaboration with information on live births and neonatal deaths that were collected from 1 January 2000 with high completeness (at least 80%) for birthweight, gestational age and sex variables. We excluded individual birth records missing either birthweight, gestational age, sex or with a gestational age below 22^+0^ weeks or more than 44^+6^ weeks of gestation, for which it was not possible to assess size for gestational age. Birth records with implausible birthweights (<250 g or ≥6500 g) or implausible combinations of birthweight and gestational age (defined as birthweight ±5 standard deviations from the mean birthweight at each completed week of gestational age) were also excluded. We evaluated the plausibility of these data sets by comparing the difference between the calculated neonatal mortality rate (NMR) in the data set and the nationally reported NMR for the same year (Table S4). We excluded specific years of data collection for which we could not undertake this assessment because of the lack of availability of nationally reported neonatal mortality (e.g. Lebanon 2002–2016 and 2018–2019) or when we were not able to calculate NMR due to small or masked cells (e.g. Northern Ireland; Figure 1).

### 2.3 Data quality

Those who die in the early neonatal period, many of whom are the smallest, are most likely to have missing variables or be missing from data sets entirely. Therefore, to assess the potential impact of this we calculated the percentage of missing variables (birthweight, gestational age and sex) for included country‐years (Table S5). Table S6 describe the metadata and reporting criteria for the very preterm for each of the 15 countries. We also assessed the impact of registration practices on mortality estimates for each country and region by calculating gestation‐specific NMR among babies born between 22 and 32 weeks of gestation (Figures S1 and S2) and by comparing the NMR for babies born at or after 22 weeks versus born at or after 24 weeks of gestation (Table S7).

### 2.4 Exposure definitions

Each baby was categorised based on strata of birthweight (objective 1), gestational age (objective 2) and newborn types (objective 3) combining gestational age, size for gestational age and sex using a modified version of the INTERGROWTH‐21st international newborn size standards extended to include from 22^+0^ to 44^+6^ weeks of gestation.^21^, ^22^, ^23^

For objective 1, all live births with birthweight recorded were included in the analysis using strata of 500‐g increment (e.g. <1000, 1000–1500 g, etc.), and a reference group between 2500 and 4000 g. For objective 2, live births at 22^+0^ weeks or later were included in analyses using classification for preterm birth based on severity (e.g. extremely preterm: <28^+0^ weeks, very preterm: 28^+0^–31^+6^ weeks, moderate preterm: 32^+0^–33^+6^ weeks, late preterm: 34^+0^–36^+6^ weeks, post‐term: ≥42^+0^ weeks of gestation) with term births as a reference group (37^+0^–41^+6^ completed weeks of gestation). For objective 3, we categorised every newborn based on gestational age (preterm birth <37^+0^ completed weeks [PT] or term ≥37^+0^ weeks [T]), and size for gestational age (defined as SGA <10th centile; LGA >90th centile; or AGA between 10th and 90th centile). We created a mutually exclusive set of six newborn types: one reference group T + AGA; four with small babies (PT + SGA, PT + AGA, PT + LGA, T + SGA) and one with large babies (T + LGA; Figure S3a).

Also, we performed a sensitivity analysis combining gestational age (PT versus T), size (SGA, AGA, LGA) and adding birthweight (LBW <2500 g or nonLBW ≥2500 g) to assess a secondary set of ten newborn types including one reference group T + AGA + nonLBW; eight including small babies (T + AGA + LBW, T + SGA + nonLBW, T + SGA + LBW, PT + LGA + nonLBW, PT + LGA + LBW, PT + AGA + nonLBW, PT + AGA + LBW, PT + SGA + LBW) and one with large babies (T + LGA + nonLBW; Figure S3b).

### 2.5 Data analysis

The relative risk of an event (death) is the likelihood of its occurrence among babies within the risk groups (gestational age, birthweight or neonatal types) compared with a reference group, and the population attributable risk is the percentage of cases (deaths) that would be attributable to the risk factor of interest (gestational age or birthweight groups or newborn types).^24^ Among the included newborn records, we calculated:

- Prevalence = the number of live births reported in each group of interest/total number of live births.
- Risk (neonatal mortality rate) = the number of live births that experienced the event (neonatal death)/total number of live births exposed to the risk of that event per 1000.
- Risk ratio = risk (neonatal mortality rate) in each group of interest/risk (neonatal mortality rate) in the reference group.
- Population attributable risk = the prevalence multiplied by the relative risk in each group of interest/the sum of the prevalence multiplied by the relative risk of all categories in the population of interest.

Each country team analysed their data sets using standardised stata (statacorp, College Station, tx, usa), R or SAS (SAS Institute, Cary, NC, USA) programming codes developed centrally by the London School of Hygiene & Tropical Medicine (LSHTM). Standard summary results tables were shared in a hub administered online by LSHTM.

## 3 RESULTS

Information on 144 country‐years including 125.5 million live births and 576 018 deaths collected between 2000 and 2020 in 15 countries was included for analysis (Figure 1 and Table S4). Overall, NMR was highest in Brazil (7.4) and Mexico (6.1) with most countries reporting NMR lower than 5 deaths per 1000 live births (Lebanon: 4.5, the USA: 4.1, the Netherlands: 3.7, Qatar: 3.1, Canada: 2.3, Denmark: 2.4, England & Wales: 2.2, Scotland: 2.4, Czech Republic: 1.6, Sweden: 1.3, Uruguay: 1.3, and Estonia: 1.2).

### 3.1 Objective 1: Neonatal mortality risk associated with birthweight categories

**FIGURE 2:**
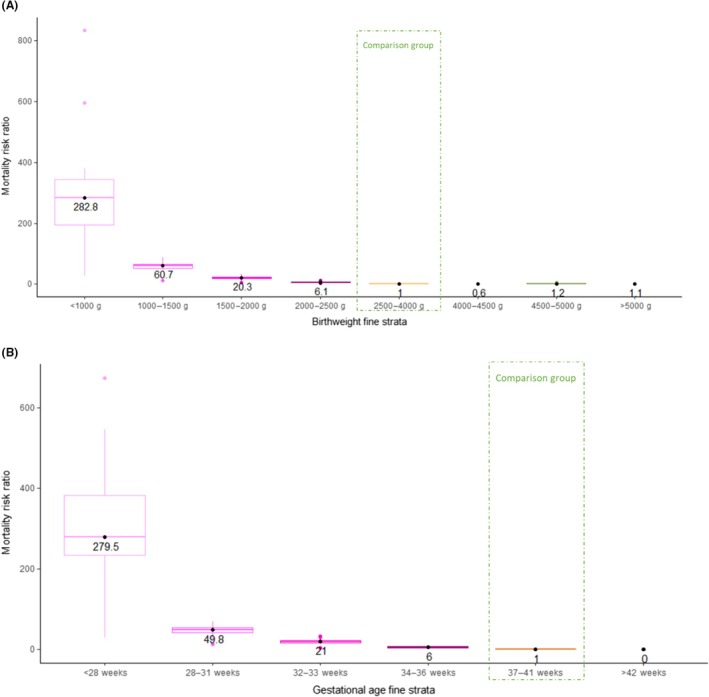
Mortality risk ratios by birthweight and gestational age, for 15 countries from 2000 to 2020. (A) Live births with birthweight recorded (*n* = 125 503 910). (B) Live births with gestational age recorded (*n* = 122 951 125). Each point represents the mortality risk ratio. Box plots summarise median values and interquartile ranges (25th and 75th centiles).

Mortality was highest among the smallest babies: the median relative risk (RR) of neonatal mortality was 280‐fold for babies less than 1000 g (median RR 282.8, interquartile range [IQR] 194.7–342.8), 60‐fold for those between 1000 and 1500 g (median RR 60.7, IQR 51.0–66.2), 20‐fold for those between 1500 and 2000 g (median RR 20.3, IQR 17.4–23.8) and 6‐fold (median RR 6.1, IQR 5.6–7.7) for babies between 2000 and 2500 g, compared with those between 2500 and 4000 g (Table 2 and Figure 2A).

**TABLE 2:** Number of live births, deaths, median prevalence, neonatal mortality rate, relative risk and population attributable risk (PAR) in 15 countries, results by fine strata of birthweight, gestational age and six newborn types.

Categories	Live births	Deaths	Prevalence	NMR	Relative risk	PAR (%)
	Number (%)	Number (%)	Median (IQR)	Median (IQR)	Median (IQR)	Median (IQR)
Birthweight, fine strata in g						
<1000 g	806 220	298 351	0.4	286.3	282.8	41.2
	0.6	51.8	(0.3–0.5)	(149.4–359.1)	(194.7–342.8)	(30.0–50.4)
1000–1500 g	889 149	53 206	0.6	38.7	60.7	11.8
	0.7	9.2	(0.5–0.7)	(32.8–44.5)	(51.0–66.2)	(8.1–12.9)
1500–2000 g	1 907 640	42 583	1.4	13.2	20.3	7.2
	1.5	7.4	(1.2–1.6)	(11.2–16.5)	(17.4–23.8)	(6.4–9.5)
2000–2500 g	6 282035	43 319	4.4	4.7	6.1	6.1
	5.0	7.5	(4.0–5.0)	(3.4–5.6)	(5.6–7.7)	(5.7–8.6)
2500–4000 g	105 710 403	130 077	83.8	0.7	Reference	Reference
	84.2	22.6	(80.2–85.2)	(0.4–0.8)		
4000–4500 g	8 532 051	6308	8.4	0.5	0.6	−1.1
	6.8	1.1	(5.6–11.3)	(0.3–0.6)	(0.6–0.8)	(−1.7 to −0.4)
4500–5000 g	1 233 821	1506	1.1	0.7	1.2	0.1
	1.0	0.3	(0.7–1.8)	(0.7–1.0)	(1.0–2.2)	(0.0–0.3)
>5000 g	142 370	673	0.1	0.9	1.5	0.1
	0.1	0.1	(0.1–0.2)	(0.0–3.2)	(0.0–4.1)	(0.0–0.1)
Gestational age, fine strata in completed weeks						
<28	661 172	197 292	0.4	273.2	279.5	40.2
	0.5	42.1	(0.3–0.5)	(190.0–322.7)	(234.2–388.5)	(30.8–43.7)
28–31	1 129 628	56 329	0.7	32.4	49.8	10.9
	0.9	12.0	(0.7–0.9)	(22.8–38.7)	(41.7–54.9)	(9.5–13.5)
32–33	1 494 543	27 192	0.9	13.6	21.0	5.7
	1.2	5.8	(0.9–1.1)	(11.8–17.3)	(17.0–22.6)	(5.1–7.5)
34–36	8 786 215	51 030	5.5	4.3	6.0	9.3
	7.1	10.9	(5.0–7.0)	(2.6–5.9)	(4.7–7.1)	(8.3–10.4)
37–42	110 525 200	135 690	92.3	0.7	Reference	Reference
	89.9	29.0	(90.4–93.0)	(0.4–0.8)		
>42	354 266	1043	0	0	0	0.0
	0.3	0.2	(0–0)	(0–1.6)	(0–1.5)	(0.0–0.0)
Newborn types						
PT + SGA	909 260	61 109	0.7	32.0	67.2	10.5
	0.7	13.0	(0.6–0.8)	(24.1–50.7)	(45.6–73.9)	(8.8–12.1)
PT + AGA	8 906 867	233 632	6.0	20.9	34.3	53.7
	7.2	49.8	(5.6–7.1)	(15.9–25.0)	(23.9–37.5)	(44.5–54.9)
PT + LGA	2 251 550	38 166	1.0	16.7	28.3	7.5
	1.8	8.1	(0.8–1.3)	(13.8–20.2)	(18.4–32.3)	(6.3–8.3)
T + SGA	5 706 866	33 978	4.1	3.5	5.4	4.3
	4.6	7.2	(3.2–5.4)	(2.6–4.6)	(4.4–6.3)	(3.3–5.7)
T + AGA	84 137 711	87 500	68.8	0.6	Reference	Reference
	68.4	18.6	(67.3–70.9)	(0.4–0.7)		
T + LGA	20 016 260	14 852	18.2	0.5	0.8	−1.1
	17.1	3.2	(13.5–22.0)	(0.3–0.5)	(0.7–0.8)	(−0.7 to −1.4)

At the population level, most neonatal deaths were LBW babies, particularly babies born below 1000 g (median PAR% 41.2, IQR 30.0–50.4), followed by those between 1000 and 1500 g (median PAR% 11.8, IQR 8.1–12.9), 1500 and 2000 g (median PAR% 7.2, IQR 6.4–9.5) and 2000–2500 g (median PAR% 6.1, IQR 5.7–8.6; Table 2).

For bigger babies, the median relative risk among those born above 4500 g was 1.2 (IQR 1.0–2.2) when compared with the reference group between 2500 and 4000 g. This measure showed greater variability among the group heavier than 5000 g (median 1.5, IQR 0.0–4.1) with higher relative mortality risk in Canada (RR 18.8, 95% CI 14.3–24.8), Australia (RR 17.1, 95% CI 8.5–34.4) and Brazil (RR 6.9, 95% CI 6.2–7.8), no evidence of an increased risk in Denmark, Scotland, Sweden, England & Wales, and zero observed deaths in Czech Republic, Estonia, Lebanon, Mexico, Qatar and Uruguay (Figure 2A and Table S8).

### 3.2 Objective 2: Neonatal mortality risk associated with gestational age

Extremely preterm babies, born before 28 weeks, had the highest neonatal mortality rate (median 273.2 deaths per 1000 live births, IQR 190.0–322.7), followed by those very preterm babies born from 28 to 31 weeks (median 32.4, IQR 22.8–38.7), moderate preterm babies born from 32 to 33 weeks (median 13.6, IQR 11.8–17.3) and late preterm, born from 34 to 36 weeks (median 4.3, IQR 2.6–5.9) (Table 2 and Table S9).

The risk of dying increased with lower gestational age; babies born extremely preterm had an almost 300‐fold increased risk (median RR 279.5, IQR 234.2–388.5) compared with babies born between 37 and 42 completed weeks as a reference group, followed by those very preterm (median RR 49.8, IQR 41.7–54.9), moderate preterm (median RR 21.0, IQR 17.0–22.6) and late preterm (median RR 6.0, IQR 4.7–7.1; Table 2, Figure 2B and Table S9).

Across the 15 countries, most neonatal deaths were attributed to babies born below 28 weeks (median PAR% 40.2, IQR 30.8–43.7), followed by the group between 28 and 31 weeks (median PAR% 10.9, IQR 9.5–13.5), 34 to 36 weeks (median PAR% 9.3, IQR 8.3–10.4) and 32 to 33 weeks (median PAR% 5.7, IQR 5.1–7.5; Table 2).

### 3.3 Objective 3: Neonatal mortality risk associated with newborn types

Applying the six newborn types, reported neonatal deaths were more common among PT + SGA live births (median mortality rate 32.0 deaths per 1000 live births, IQR 24.1–50.7), followed by PT + AGA (median mortality rate 20.9, IQR 15.9–25.0) and PT + LGA (median mortality rate 16.7 deaths per 1000 live births, IQR 13.8–20.2), T + SGA (median mortality rate 3.5 deaths per 1000 live births, IQR 2.6–4.6), T + AGA (median mortality rate 0.6, IQR 0.4–0.7), and T + LGA (median mortality rate 0.5 per 1000 live births, IQR 0.3–0.5).

**FIGURE 3:**
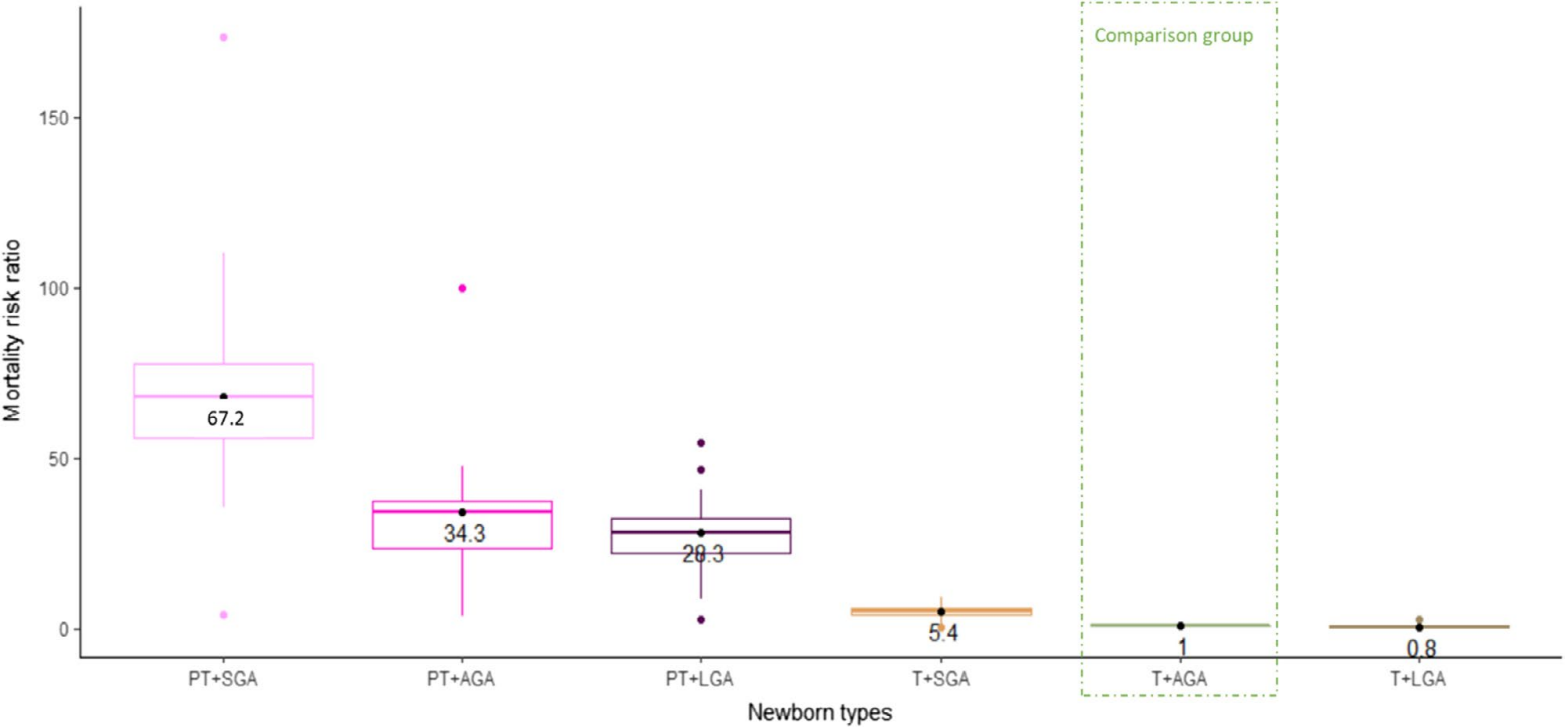
Mortality risk ratios by six newborn types, for 15 countries from 2000 to 2020. Live births with newborn types assessed (*n* = 122 928 744). Each point represents the relative risk ratio by country. Box plots summarise the median values and interquartile ranges (25th and 75th centiles).

The highest relative risk was around 70‐fold for PT + SGA (median RR 67.2, IQR 45.6–73.9), followed by PT + AGA (median RR 34.3, IQR 23.9–37.5), PT + LGA (median RR 28.3, IQR 18.4–32.3) and T + SGA (median RR 5.4, IQR 4.4–6.3) when compared with the reference category T + AGA (Table 2, Figure 3 and Table S10).

At population level, most neonatal deaths were attributed to PT + AGA (median PAR% 53.7, IQR 44.5–54.9), PT + SGA (median PAR% 10.5, IQR 8.8–12.1), PT + LGA (median PAR% 7.5, IQR 6.3–8.3) and T + SGA (median PAR% 4.3, IQR 3.3–5.7; Table 2 and Table S10).

A sensitivity analysis considering ten newborn types instead of six, showed that the highest relative risks were among types with the co‐existence of preterm and LBW such as those PT + LGA + LBW (median RR 114.0, IQR 102.6–139.5), PT + SGA + LBW (median RR 66.8, IQR 45.3–76.7) and PT + AGA + LBW (median RR 54.3, IQR 44.1–60.6). The median mortality risk ratio for preterm and non‐LBW types was ten‐fold (median 10.2, IQR 7.7–13.2) for PT + LGA + nonLBW and four‐fold (median 4.2, IQR 3.3–5.4) for PT + AGA + nonLBW. Among the term types, the median relative risk was nine‐fold (median 9.0, IQR 7.6–13.2) among T + SGA + LBW, three‐fold (median 3.1, IQR 1.8–4.3) for T + AGA + LBW and 2.6‐fold (IQR 1.9–3.4) for T + SGA + nonLBW. Large babies (T + LGA + nonLBW) did not show a greater risk of dying compared with the reference group (T + AGA + nonLBW; Table S11).

## 4 DISCUSSION

### 4.1 Main findings

Our data set of more than 125.5 million live‐birth records collected in 15 countries over two decades has provided the first multi‐country estimates of mortality related to novel newborn types across regions of North America, Australia, Central Asia and Europe (ten countries), Latin America and the Caribbean (three countries), and western Asia and North Africa (two countries; Figure 1). Data quality was high at least for completeness of three core variables (birthweight, gestational age and sex; completeness ≥80%).

We found that being both preterm and SGA was the most predictive type in identifying vulnerability to neonatal mortality risk across all countries (PT + SGA median RR 67.2), followed by those PT + AGA (median RR 34.3) and PT + LGA (median RR 28.3). However, as PT + SGA has low prevalence, the PAR% is highest for PT + AGA. As both PT + AGA and PT + LGA had median relative risks around 30, in future, collapsing these two groups into a single ‘preterm not SGA’ group could further simplify the newborn types to only three, without losing the ability to identify neonatal mortality risk.

The four categories of preterm birth were found to be useful to identify infants at risk of neonatal death. However, neonatal mortality risk was driven particularly by lower gestational age with a clear dose–response (median RR for <28 weeks 279.5, for 28–31 weeks 49.8, for 32–33 weeks 21.0 and for 34–36 weeks 6.0). Birthweight strata also showed a dose–response, with the highest risk at the lower weights (median RR for <1000 g 282.8, for 1000–1500 g 60.7, for 1500–2000 g 20.3 and for 2000–2500 g 6.1), however, this is likely to be driven by the association between birthweight and gestational age. Given the major variation in risk by gestational age, we underline the value of considering this as a continuum, rather than a dichotomous cutoff at 37 weeks.

Mortality rates for babies born before 28 weeks varied by country, with the highest rates reported in Lebanon (542.9 deaths per 1000 babies) and Brazil (428.6 deaths per 1000 babies) and the lowest rates in Sweden (136.8 deaths per 1000 babies) and Estonia (137.1 deaths per 1000 babies). These large national variations could be reflective of true differences in population risk (e.g. higher mortality rates expected with more restrictive policies about abortions for congenital anomalies), or variations in access to high‐quality neonatal intensive care.^25^ However, it is well recognised that registration systems can selectively miss liveborn newborns at the extremes of gestational age and birthweight and international or inter‐hospital comparisons of neonatal mortality may be misleading if these biases are not considered.^26^, ^27^

Bigger babies also had an increased risk of neonatal death, as noted overall for babies born after 42 weeks in Brazil and the USA (compared with 37–42 weeks) and those born heavier than 4500 g (compared with normal birthweight). The T + LGA category did not show additional risk for early mortality. A more detailed analysis of vulnerability in LGA babies is the focus of another paper in this supplement.^28^

### 4.2 Interpretation

Our analysis uses and adapts the recently described *Lancet* Small Vulnerable Newborn classification^15^ to better delineate underlying causal pathways, identify the most vulnerable babies and target interventions. Our paper helps to inform future applications of this classification. The use of six newborn types (combining gestational age and size) versus ten (combining gestational age, size and birthweight) may be helpful for clinical practice, public health policy and research. Using the six newborn types confirmed the finding that the coexistence of preterm and SGA drives a higher mortality risk.^7^ Given that LBW is a consequence of being born preterm and/or SGA age, dropping the LBW outcome may offer a more parsimonious and still useful approach to identifying newborns with common determinants.^29^ Given that gestational age is the main driver of neonatal mortality risk, further research could consider splitting newborn types by gestational age bands. Also, future research is needed to clarify the best category to approach the vulnerability of bigger babies, such as those above the 97th centile or post‐term.^16^, ^17^, ^18^

### 4.3 Strengths and limitations

This multi‐country collaboration has substantial strengths regarding the analysis of large national routine administrative data sets with more than 125.5 million live births and almost 600 000 neonatal deaths. These results are likely to be representative of the overall populations in these countries because these data sets included more than 80% of all live births in the country with high levels of completeness for three key variables to assess newborn types. Another strength is the use of a common international standard (INTERGROWTH‐21st) for direct comparisons among 15 countries data.

Although data quality was high in terms of completeness, there were some remaining limitations due to missing variables and record linkage quality (Tables S5 and S6). More importantly, we cannot fully account for inter‐country variability in perceived viability and reporting of very preterm babies (Table S6), which still poses challenges to international comparisons of neonatal mortality.^26^, ^27^ Variability in the registration of very premature babies was particularly noted among babies born between 22^+0^ and 23^+6^ weeks (Figures S1 and S2), impacting the ranking of national mortality rates for babies born at or after 22 versus at or after 24 completed weeks of gestation (Table S7). Another limitation is the lack of confirmation of the method for gestational age estimation, this may drive potential misclassifications on size for gestational age as some data sets only provided gestational age data in completed weeks and not exact days.

In addition, no eligible data sets were identified from Sub‐Saharan Africa or southern Asia where more than 80% of all neonatal deaths occur and where neonatal survival progress is needed the most.^3^ To seek to close this gap, the Vulnerable Newborn Measurement Collaborative group have analysed sub‐national data from research studies in these regions.^30^ This paper focuses only on neonatal deaths following live birth, but stillbirths are presented in another paper in this series.^31^

Many important research gaps are highlighted by this work. Although accurate gestational age assessment is widely available in countries participating in this study, such information is more limited in many high‐burden settings, which could limit the applicability of these newborn types in these settings. Innovative bedside tools to assess both gestational age and size‐for‐gestational age could help to target interventions and improve care and survival. Cohort analyses using these types would be valuable to provide more granular information on medium to long‐term risk of non‐fatal life‐course outcomes including non‐communicable conditions than traditional analyses based on LBW alone.

This novel multi‐country analysis is based on large and nationwide data sets with 125.5 million live births and more than half a million neonatal deaths collected in 15 high‐ and upper‐middle‐income countries. These six newborn types were found to be predictive of those most vulnerable to neonatal mortality and could be useful clinically to identify newborn vulnerability. Our analysis underlines again the large burden driven by preterm birth, with the greatest risk being PT + SGA and the largest population‐attributable impact being PT + AGA. The use of these newborn types could potentially help research studies to better delineate underlying causal pathways, rather than a focus on LBW dichotomous cutoffs, and accelerate progress for the prevention of 15 million preterm births per year.

## AUTHOR CONTRIBUTIONS

The Vulnerable Newborn collaborative was planned by JEL, REB and co‐ordinated by JY. This analysis was designed by HB and EOO with JEL. YBO, JY and all authors contributed to the study protocol, with inputs from the wider Vulnerable Newborn Measurement Collaboration. Country data analyses were undertaken and revised by AG, VF, ESP, MLB, SL, QW, PV, JJ, EHP, HTS, LS, LA, KAY, AAB, AB, LB, AED, FA, TOO, NR, JS, LKS, ESD, EL, NR, RW, KM, IP, and GP. Pooled analysis was undertaken by LSI with EB and EOO. The manuscript was drafted by LSI with HB, EOO and JEL. All authors helped revise the manuscript. All authors reviewed and agreed on the final version.

## FUNDING INFORMATION

This analysis was funded by Children's Investment Fund Foundation, prime grant 1803‐02535. The funders had no role in the study design, data collection, analysis or interpretation of the paper. ESP and MLB received funding from Wellcome Trust UK (202912/B/16/Z). NR received funding from Swedish Research Council (VR 2979/2020). ED received funding from the MBRRACE‐UK programme.

## CONFLICT OF INTEREST STATEMENT

None declared.

## ETHICS APPROVAL

The Vulnerable Newborn Measurement Collaboration was granted ethical approval from the Institutional Review Boards of the London School of Hygiene & Tropical Medicine (ref: 22858, date of approval: 17 May 2021) and Johns Hopkins Bloomberg School of Public Health (IRB No: 16439, date of approval 8 May 2021). All the 15 country teams had ethical approval for use of data or exemptions based on the current remit.

## DISCLOSURES

We are grateful to Consultative Council on Obstetric and Paediatric Mortality and Morbidity (CCOPMM) for providing access to the data used for this project and for the assistance of the staff at Safer Care Victoria. The conclusions, findings, opinions and views or recommendations expressed in this paper are strictly those of the author(s). They do not necessarily reflect those of CCOPMM. We would like to acknowledge and thank the NT Perinatal Data team for access to the Northern Territory perinatal data collection. Australian data were provided to the CRE team and international team with small numbers, those less than 5, suppressed.

## Supporting information


Appendix S1.

## Data Availability

Data sharing and transfer agreements were jointly developed and signed by all collaborating partners. All data used in these analyses are available in the Supplementary Information. The pooled aggregate data will be available at https://doi.org/10.17037/DATA.00003095 at the time of publication with the exception of those from countries where data sharing is not permitted.

## APPENDIX 1

### National Collaborative Group for Vulnerable Newborn Mortality

Australia: Vicki Flenady; Adrienne Gordon; Kara Warrilow; Harriet Lawford. Brazil: Enny S. Paixao; Mauricio Lima Barreto; Ila Rocha Falcao. Canada: Sarka Lisonkova; Qi Wen. Czech Republic: Petr Velebil; Jitka Jírová. Denmark: Erzsébet Horváth‐Puhó, Henrik T. Sørensen. Estonia: Luule Sakkeus; Liili Abuladze. Lebanon: Khalid A. Yunis; Ayah Al Bizri; Pascale Nakad. Mexico: Lorena Suárez‐Idueta; Arturo Barranco Flores; Jesus Felipe Gonzalez Roldan; Sonia Lopez Alvarez. Netherlands: Lisa Broeders; Aimée E. van Dijk. Qatar: Fawziya Alyafei; Mai AlQubaisi; Tawa O. Olukade; Hamdy A. Ali; Mohamad Rami Alturk. Sweden: Neda Razaz; Jonas Söderling. UK_England and Wales: Lucy K. Smith; Bradley N. Manktelow; Ruth J. Matthews; Elizabeth Draper; Alan Fenton; Jennifer J. Kurinczuk. UK_Northern Ireland: Estelle Lowry; Neil Rowland. UK_Scotland: Rachael Wood; Celina Davis; Kirsten Monteath; Samantha Clarke. Uruguay: Isabel Pereyra, Gabriella Pravia. USA: Sarka Lisonkova; Qi Wen.

### Vulnerable newborn Measurement Core Group

LSHTM: Joy E. Lawn; Hannah Blencowe; Eric Ohuma; Yemisrach B. Okwaraji; Judith Yargawa; Ellen Bradley. JHU: Robert E. Black; Joanne Katz; Daniel J. Erchick; Elizabeth A. Hazel; Michael Diaz; Anne C. C. Lee.
