# Supplementary material for: Neonatal mortality risk for vulnerable newborn types in 15 countries using 125.5 million nationwide birth outcome records, 2000–2020
Source: BJOG. 2023 May 8;132(Suppl 8):S37–47. doi: 10.1111/1471-0528.17506 (PMC12678064; doi:10.1111/1471-0528.17506)
Supplement: Supplementary file 1 — Appendix S1. [file BJO-132-S37-s001.docx]

**SUPPLEMENT TITLE**: Vulnerable Newborn multi-country analyses related to preterm births and small-for-gestational age

**PAPER TITLE**

Neonatal mortality risk for vulnerable newborn types in 15 countries using 125.5 million nationwide birth outcome records from 2000 to 2020

**PAPER RUNNING TITLE**

Mortality risk for newborn types in 15 countries

**SUPPORTING INFORMATION**

Table of Contents

[**Table S1: RECORD guidelines checklist** 3](#_Toc131371950)

[**Table S2: Ethics approval or exemptions of Institutional Review Boards** 9](#_Toc131371951)

[**Table S3: Definitions** 10](#_Toc131371952)

[**Input data** 11](#_Toc131371953)

[**Table S4. Assessment of plausibility of joined dataset 167 country-years from 2000 to 2020, by Objective** 11](#_Toc131371954)

[**(a: records with birthweight (BW) reported, b: records with Gestational Age (GA) reported, c: Records with newborn types assessed)** 11](#_Toc131371955)

[**Table S5. Number of missing values on three core variables (birthweight, gestational age, and sex) among included country-years** 15](#_Toc131371956)

[**Table S6. Summary of metadata** 16](#_Toc131371957)

[**Figure S1. Neonatal mortality NMRs by gestational age among very preterm births (22 to 32 completed weeks) in 15 countries** 18](#_Toc131371958)

[**Figure S2. Neonatal mortality NMRs by gestational age among very preterm births (22 to 32 completed weeks) by region** 18](#_Toc131371959)

[**Table S7. Ranking of neonatal mortality NMRs comparing two groups of babies (≥22 weeks and ≥24 weeks) in 15 countries** 19](#_Toc131371960)

[**Figure S3. Overview of Vulnerable newborn types based on gestational age, size for gestational age and birthweight.** 20](#_Toc131371961)

[**Additional results:** 22](#_Toc131371962)

[**Table S8. Neonatal mortality NMR, population attributable risk, and crude relative risk of neonatal mortality for birthweight fine strata (reference 2,500g to 4,000g), by country** 22](#_Toc131371963)

[**Table S9. Neonatal mortality NMR, population attributable risk, and crude relative risk of neonatal mortality for gestational age fine strata (reference 37 to 42 completed weeks), by country** 26](#_Toc131371964)

[**Table S10. Neonatal mortality NMR, population attributable risk, and crude relative risk of neonatal mortality for 6 newborn types (reference T+AGA), by country** 29](#_Toc131371965)

[**Table S11. Neonatal mortality NMR, population attributable risk, and crude relative risk of neonatal mortality for 10 newborn types (reference T+AGA+nonLBW), by country** 32](#_Toc131371966)

[**Additional references** 37](#_Toc131371967)

# **Table S1: RECORD guidelines checklist**

**The reporting of studies conducted using observational routinely collected data**

|  | **#** | **STROBE items** | **Location** | **RECORD items** | **Location in manuscript where items are reported** |
| --- | --- | --- | --- | --- | --- |
| **Title and abstract** | | | | | |
|  | 1 | (a) Indicate the study’s design with a commonly used term in the title or the abstract (b) Provide in the abstract an informative and balanced summary of what was done and what was found |  | RECORD 1.1: The type of data used should be specified in the title or abstract. When possible, the name of the databases used should be included.  RECORD 1.2: If applicable, the geographic region and timeframe within which the study took place should be reported in the title or abstract.  RECORD 1.3: If linkage between databases was conducted for the study, this should be clearly stated in the title or abstract. | *Title: “**Neonatal mortality risk for vulnerable newborn types in 15 countries using 125.5 million nationwide birth outcome records, 2000 to 2020”* |
|  | | | | | |
| Background rationale | 2 | Explain the scientific background and rationale for the investigation being reported |  |  | Introduction  (Paragraphs 1-2) |
| Objectives | 3 | State specific objectives, including any prespecified hypotheses |  |  | Introduction  (Paragraph 3) |
|  | | | | | |
| Study Design | 4 | Present key elements of study design early in the paper |  |  | Methods  (Paragraph 1) |
| Setting | 5 | Describe the setting, locations, and relevant dates, including periods of recruitment, exposure, follow-up, and data collection |  |  | Methods  (Paragraphs 1-2) |
| Participants | 6 | *(a) Cohort study* - Give the eligibility criteria, and the sources and methods of selection of participants. Describe methods of follow-up  *Case-control study* - Give the eligibility criteria, and the sources and methods of case ascertainment and control selection. Give the rationale for the choice of cases and controls  *Cross-sectional study* - Give the eligibility criteria, and the sources and methods of selection of participants  *(b) Cohort study* - For matched studies, give matching criteria and number of exposed and unexposed  *Case-control study* - For matched studies, give matching criteria and the number of controls per case |  | RECORD 6.1: The methods of study population selection (such as codes or algorithms used to identify subjects) should be listed in detail. If this is not possible, an explanation should be provided.  RECORD 6.2: Any validation studies of the codes or algorithms used to select the population should be referenced. If validation was conducted for this study and not published elsewhere, detailed methods and results should be provided.  RECORD 6.3: If the study involved linkage of databases, consider use of a flow diagram or other graphical display to demonstNMR the data linkage process, including the number of individuals with linked data at each stage. | Methods  (Paragraphs 4)  under the subheading Inclusion and Exclusion criteria  Figure 1. Flowchart |
| Variables | 7 | Clearly define all outcomes, exposures, predictors, potential confounders, and effect modifiers. Give diagnostic criteria, if applicable. |  | RECORD 7.1: A complete list of codes and algorithms used to classify exposures, outcomes, confounders, and effect modifiers should be provided. If these cannot be reported, an explanation should be provided. | Methods  under the subheading exposure definitions |
| Data sources/ measurement | 8 | For each variable of interest, give sources of data and details of methods of assessment (measurement).  Describe comparability of assessment methods if there is more than one group |  |  | Methods  under the subheading exposure definitions |
| Bias | 9 | Describe any efforts to address potential sources of bias |  |  | Quality assessment described Methods under the subheading Data quality (Supplementary material S4b, S4c, S4d, S4f) |
| Study size | 10 | Explain how the study size was arrived at |  |  | Methods under the subheading Inclusion and exclusion criteria. Flowchart Figure 1 |
| Quantitative variables | 11 | Explain how quantitative variables were handled in the analyses. If applicable, describe which groupings were chosen, and why |  |  | Methods under the subheading exposure definitions |
| Statistical methods | 12 | (a) Describe all statistical methods, including those used to control for confounding  (b) Describe any methods used to examine subgroups and interactions  (c) Explain how missing data were addressed  (d) *Cohort study* - If applicable, explain how loss to follow-up was addressed  *Case-control study* - If applicable, explain how matching of cases and controls was addressed  *Cross-sectional study* - If applicable, describe analytical methods taking account of sampling stNMRgy  (e) Describe any sensitivity analyses |  |  | Methods under the subheading data analysis |
| Data access and cleaning methods |  | .. |  | RECORD 12.1: Authors should describe the extent to which the investigators had access to the database population used to create the study population.  RECORD 12.2: Authors should provide information on the data cleaning methods used in the study. | Methods under the subheading data analysis |
| Linkage |  | .. |  | RECORD 12.3: State whether the study included person-level, institutional-level, or other data linkage across two or more databases. The methods of linkage and methods of linkage quality evaluation should be provided. | Supplementary table S4c |
| **Results** | | | | | |
| Participants | 13 | (a) Report the numbers of individuals at each stage of the study (*e.g.*, numbers potentially eligible, examined for eligibility, confirmed eligible, included in the study, completing follow-up, and analysed)  (b) Give reasons for non-participation at each stage.  (c) Consider use of a flow diagram |  | RECORD 13.1: Describe in detail the selection of the persons included in the study (*i.e.,* study population selection) including filtering based on data quality, data availability and linkage. The selection of included persons can be described in the text and/or by means of the study flow diagram. | Results (Paragraph 1 and Figure 1) |
| Descriptive data | 14 | (a) Give characteristics of study participants (*e.g.*, demographic, clinical, social) and information on exposures and potential confounders  (b) Indicate the number of participants with missing data for each variable of interest  (c) *Cohort study* - summarise follow-up time (*e.g.*, average and total amount) |  |  | Supplementary material S4b |
| Outcome data | 15 | *Cohort study* - Report numbers of outcome events or summary measures over time  *Case-control study* - Report numbers in each exposure category, or summary measures of exposure  *Cross-sectional study* - Report numbers of outcome events or summary measures |  |  | Results (Paragraph 2-12, Figures 2 and 3, and Supplementary material S5a, S5b, S5c) |
| Main results | 16 | (a) Give unadjusted estimates and, if applicable, confounder-adjusted estimates and their precision (e.g., 95% confidence interval). Make clear which confounders were adjusted for and why they were included  (b) Report category boundaries when continuous variables were categorized  (c) If relevant, consider translating estimates of relative risk into absolute risk for a meaningful time period |  |  | Results (Paragraph 2-12, Figures 2 and 3, and Supplementary material S5a, S5b, S5c) |
| Other analyses | 17 | Report other analyses done—e.g., analyses of subgroups and interactions, and sensitivity analyses |  |  | Results (Paragraph 12) |
|  | | | | | |
| Key results | 18 | Summarise key results with reference to study objectives |  |  | Discussion under the subheading main findings |
| Limitations | 19 | Discuss limitations of the study, taking into account sources of potential bias or imprecision. Discuss both direction and magnitude of any potential bias |  | RECORD 19.1: Discuss the implications of using data that were not created or collected to answer the specific research question(s). Include discussion of misclassification bias, unmeasured confounding, missing data, and changing eligibility over time, as they pertain to the study being reported. | Discussion under the subheading strengths and limitations |
| Interpretation | 20 | Give a cautious overall interpretation of results considering objectives, limitations, multiplicity of analyses, results from similar studies, and other relevant evidence |  |  | Discussion under the subheading interpretation |
| Generalisability | 21 | Discuss the generalisability (external validity) of the study results |  |  | Discussion under the subheading strengths and limitations |
|  | | | | | |
| Funding | 22 | Give the source of funding and the role of the funders for the present study and, if applicable, for the original study on which the present article is based |  |  | The source of founding is included in the Abstract and the funding role is described at the end of the manuscript under the subheading Funding role |
| Accessibility of protocol, raw data, and programming code |  | .. |  | RECORD 22.1: Authors should provide information on how to access any supplemental information such as the study protocol, raw data, or programming code. | Under the subheading Availability of data and material |

Source ^1^

# **Table S2: Ethics approval or exemptions of Institutional Review Boards**

| **Country of origin for data** | **Institutional Review Board(s) or data access provider** | **Ref/Number** | **Date of approval** |
| --- | --- | --- | --- |
| London School of Hygiene & Tropical Medicine (LSHTM) | LSHTM - Observational / Interventions Research Ethics Committee | 22858 | 17^th^ May 2021 |
| Australia | Australian Institute of Health and Welfare Ethics Committee | EO2018/2/451 | 4^th^ May 2021 |
| Brazil | Federal University of Bahia’s Institute of Public Health Ethics Committee | 18022319.4.0000.5030 | 3^rd^ September 2019 |
| Canada | UBC C&W Research Ethics Board | H21-00653 | 31^st^ March 2021 |
| Estonia | Ethics Committee of National Institute for Health Development | 770 | 9^th^ August 2021 |
| Lebanon | Institutional Review Board, American University of Beirut | PED.KY.01 | 13^th^ July 2021 |
| Mexico | Centre of Investigation in Health Sciences, Anahuac University, Mexico | 202214 | 31^st^ March 2022 |
| Qatar | Medical Research Center, Hamad Medical Corporation, Doha-Qatar | MRC-01-21-277 | 25^th^ April 2021 |
| UK_England and Wales | 1. National Information Governance Board  2. Confidentiality Advisory Group of the Health Research Authority  3. Health & Social Care Information Centre (HSCIC), Data Access Advisory Group | 1. ECC 5-05 (f)/2012  2. 15/CAG/0119  3. DARS-NIC-359651-H3R1P-v5.2. | 1. 10^th^ October 2012  2. 1^st^ May2015 |
| UK_Scotland | Public Health Scotland | 20210218-VulnerableNewbornMeasurement | 30^th^ March 2021 |
|  | | | |
| **Exemptions (*e.g., IRB approval not required for public or aggregate data, existing ethics approval in place, etc)*** | | | |
| Czech Republic | | | |
| Denmark | | | |
| Netherlands | | | |
| Sweden | | | |
| Uruguay | | | |
| USA publicly available data from https://www.cdc.gov/nchs/data_access/Vitalstatsonline.htm | | | |

# **Table S3: Definitions**

Consistent with the International Classification of Disease 10^th^ and 11^th^ revisions ^2, 3^

INTERGROWTH-21st international standards ^4, 5^

| **Definitions** |  |
| --- | --- |
| Livebirth | Is the complete expulsion or extraction from a woman of a fetus, irrespective of the duration of the pregnancy, which, after such separation, shows signs of life |
| Birthweight | Is defined as the weight of the fetus or newborn obtained immediately after birth. For livebirths, measurement of birthweight within the first hour of life before significant postnatal weight loss has occurred is preferable. If the birth weight was measured repeatedly, the median value will be used. Birthweight can be measured using digital or analogue scales. For the purposes of this work, weights of newborn taken at ≥72 hours after birth will be excluded. |
| Gestational age | The duration of gestation measured from the first day of the last menstrual period (LMP). Gestational age will be analysed in days where possible. Gestational age measured by LMP, early pregnancy ultrasound or best obstetric estimate (BEO) will be included |
| Neonatal death | A neonatal death is defined as a death during the first 28 days after live birth (days 0-27). An early neonatal death is a death during the first 7 days after live birth (days 0 – 6), a late neonatal death is a death day 7 – 27 after a livebirth. |
| **Calculated variables** |  |
| Preterm birth | A birth before 37 completed weeks of gestation (or before 259 days of gestation) as measured from the first day of the last menstrual period (LMP) or by early ultrasound. |
| Term birth | A birth from 37 completed weeks of gestation as measured from the first day of the last menstrual period (LMP) or by early ultrasound. |
| Low birthweight | A birth with birthweight of less than 2500grams |
| Non-Low birthweight | A birth with birthweight of ≥2500grams |
| Small for gestational age | A birth with a birthweight for gestational age and sex of <10th centile according to INTERGROWTH-21st international standards |
| Appropriate for gestational age | A birth with a birthweight for gestational age and sex from 10th to 90th centiles according to INTERGROWTH-21st international standards |
| Large for gestational age | A birth with a birthweight for gestational age and sex of >90th centile according to INTERGROWTH-21st international standards |

# **Input data**

# **Table S4. Assessment of plausibility of joined dataset 167 country-years from 2000 to 2020, by Objective**

# **(a: records with birthweight (BW) reported, b: records with Gestational Age (GA) reported, c: Records with newborn types assessed)**

Calculated Neonatal Mortality NMR (NMR): the number of persons who experienced the event (neonatal death: deaths that occurred from day 0 to 27) divided by the number of livebirths per 1000 calculated from the national dataset

Reported NMR: NMR reported by the country to the United Nations Inter-Agency Group for Child Mortality Estimation (UNIGME) ^6^

Difference= calculated NMR – Reported NMR

| **Country** | **Year** | **NMR Reported to UNIGME** | **a. Records with birthweight reported** | | | | **b. Records with gestational age reported** | | | | **c. Records with newborn types assessed** | | | |
| --- | --- | --- | --- | --- | --- | --- | --- | --- | --- | --- | --- | --- | --- | --- |
|  |  |  | **Livebirths** | **Deaths** | **NMR** | **Difference** | **Livebirths** | **Deaths** | **NMR** | **Difference** | **Livebirths** | **Deaths** | **NMR** | **Difference** |
| Australia | 2016 | 2.3 | 312,554 | 736 | 2.4 | 0.1 | 312,268 | 570 | 1.8 | -0.4 | 312,126 | 572 | 1.8 | -0.4 |
| Australia | 2017 | 2.4 | 303,382 | 739 | 2.4 | 0 | 303,148 | 551 | 1.8 | -0.6 | 303,092 | 532 | 1.8 | -0.7 |
| Australia | 2018 | 2.3 | 300,738 | 635 | 2.1 | -0.2 | 300,688 | 532 | 1.8 | -0.6 | 300,550 | 509 | 1.7 | -0.6 |
| Australia | 2019 | 2.4 | 300,654 | 624 | 2.1 | -0.3 | 300,586 | 513 | 1.7 | -0.7 | 300,404 | 483 | 1.6 | -0.8 |
| Brazil | 2011 | 9.8 | 2,909,757 | 21,552 | 7.4 | -2.4 | 1,567,290 | 10,973 | 7 | -2.8 | 1,566,290 | 10,905 | 7 | -2.8 |
| Brazil | 2012 | 9.6 | 2,903,294 | 21,437 | 7.4 | -2.2 | 2,658,567 | 18,321 | 6.9 | -2.7 | 2,657,277 | 18,224 | 6.9 | -2.7 |
| Brazil | 2013 | 9.4 | 2,901,989 | 20,978 | 7.2 | -2.2 | 2,764,608 | 18,763 | 6.8 | -2.6 | 2,763,191 | 18,737 | 6.8 | -2.6 |
| Brazil | 2014 | 9.2 | 2,977,620 | 21,038 | 7.1 | -2.1 | 2,885,796 | 19,283 | 6.7 | -2.5 | 2,884,400 | 19,272 | 6.7 | -2.5 |
| Brazil | 2015 | 9 | 3,012,086 | 21,363 | 7.1 | -1.9 | 2,926,642 | 19,564 | 6.7 | -2.3 | 2,925,494 | 19,554 | 6.7 | -2.3 |
| Brazil | 2016 | 9 | 2,857,886 | 22,341 | 7.8 | -1.2 | 2,797,095 | 20,788 | 7.4 | -1.6 | 2,796,010 | 20,600 | 7.4 | -1.6 |
| Brazil | 2017 | 8.9 | 2,920,048 | 22,805 | 7.8 | -1.1 | 2,859,648 | 21,217 | 7.4 | -1.5 | 2,858,521 | 21,016 | 7.4 | -1.5 |
| Brazil | 2018 | 8.5 | 2,943,410 | 21,641 | 7.4 | -1.1 | 2,895,362 | 20,325 | 7 | -1.5 | 2,894,256 | 20,110 | 6.9 | -1.6 |
| Canada | 2005 | 4.1 | 255,090 | 680 | 2.7 | -1.4 | 224,784 | 491 | 2.2 | -1.9 | 224,886 | 589 | 2.6 | -1.5 |
| Canada | 2006 | 3.7 | 259,971 | 600 | 2.3 | -1.4 | 252,689 | 449 | 1.8 | -1.9 | 252,780 | 567 | 2.2 | -1.5 |
| Canada | 2007 | 3.8 | 272,457 | 710 | 2.6 | -1.2 | 272,146 | 561 | 2.1 | -1.7 | 272,277 | 706 | 2.6 | -1.2 |
| Canada | 2008 | 3.7 | 282,459 | 715 | 2.5 | -1.2 | 282,145 | 548 | 1.9 | -1.8 | 282,307 | 708 | 2.5 | -1.2 |
| Canada | 2009 | 3.7 | 284,534 | 709 | 2.5 | -1.2 | 284,260 | 563 | 2 | -1.7 | 284,405 | 709 | 2.5 | -1.2 |
| Canada | 2010 | 3.9 | 281,190 | 670 | 2.4 | -1.5 | 280,904 | 509 | 1.8 | -2.1 | 281,076 | 674 | 2.4 | -1.5 |
| Canada | 2011 | 3.7 | 280,890 | 696 | 2.5 | -1.2 | 280,614 | 530 | 1.9 | -1.8 | 280,774 | 689 | 2.5 | -1.2 |
| Canada | 2012 | 3.6 | 281,802 | 656 | 2.3 | -1.3 | 281,432 | 523 | 1.9 | -1.7 | 281,561 | 652 | 2.3 | -1.3 |
| Canada | 2013 | 3.8 | 281,080 | 670 | 2.4 | -1.4 | 280,654 | 508 | 1.8 | -2 | 280,811 | 664 | 2.4 | -1.4 |
| Canada | 2014 | 3.6 | 284,452 | 636 | 2.2 | -1.4 | 283,884 | 498 | 1.8 | -1.8 | 284,015 | 632 | 2.2 | -1.4 |
| Canada | 2015 | 3.5 | 283,249 | 552 | 1.9 | -1.6 | 282,690 | 424 | 1.5 | -2 | 282,815 | 550 | 1.9 | -1.6 |
| Canada | 2016 | 3.4 | 283,931 | 537 | 1.9 | -1.5 | 283,579 | 413 | 1.5 | -1.9 | 283,703 | 539 | 1.9 | -1.5 |
| Canada | 2017 | 3.5 | 280,277 | 592 | 2.1 | -1.4 | 279,916 | 438 | 1.6 | -1.9 | 280,068 | 588 | 2.1 | -1.4 |
| Canada | 2018 | 3.4 | 276,934 | 636 | 2.3 | -1.1 | 276,649 | 486 | 1.8 | -1.6 | 276,745 | 619 | 2.2 | -1.2 |
| Canada | 2019 | 3.2 | 274,872 | 557 | 2 | -1.2 | 274,266 | 431 | 1.6 | -1.6 | 274,396 | 556 | 2 | -1.2 |
| Canada | 2020 | 3.2 | 266,267 | 561 | 2.1 | -1.1 | 265,634 | 422 | 1.6 | -1.6 | 265,768 | 558 | 2.1 | -1.1 |
| Czech Republic | 2019 | 1.6 | 112,231 | 174 | 1.6 | 0 | 109,492 | 168 | 1.5 | -0.1 | 109,492 | 168 | 1.5 | -0.1 |
| Denmark | 2000-2017 | 3.1 | 1,100,854 | 2,598 | 2.4 | -0.7 | 1,100,854 | 2,598 | 2.4 | -0.7 | 1,100,854 | 2,598 | 2.4 | -0.7 |
| Estonia | 2015 | 1.5 | 13,905 | 21 | 1.5 | 0.0 | 13,911 | 21 | 1.5 | 0.0 | 13,905 | 21 | 1.5 | 0.0 |
| Estonia | 2016 | 1.2 | 13,859 | 17 | 1.2 | 0.0 | 13,872 | 17 | 1.2 | 0.0 | 13,858 | 17 | 1.2 | 0.0 |
| Estonia | 2017 | 1.4 | 13,508 | 18 | 1.3 | 0.1 | 13,519 | 18 | 1.3 | 0.1 | 13,508 | 18 | 1.3 | 0.1 |
| Estonia | 2018 | 1 | 14,157 | 14 | 1.0 | 0.0 | 14,186 | 14 | 1.0 | 0.0 | 14,155 | 14 | 1.0 | 0.0 |
| Estonia | 2019 | 0.9 | 13,872 | 13 | 0.9 | 0.0 | 13,909 | 13 | 0.9 | 0.0 | 13,871 | 13 | 0.9 | 0.0 |
| Estonia | 2020 | 0.9 | 13,009 | 12 | 0.9 | 0.0 | 13,030 | 12 | 0.9 | 0.0 | 13,001 | 12 | 0.9 | 0.0 |
| Lebanon | 2001 | 11.5 | 10,009 | 75 | 7.5 | -4 | 9,726 | 80 | 8.2 | 3.3 | 9,700 | 72 | 7.4 | 4.1 |
| Lebanon | 2002 |  | 10,164 | 66 | 6.5 |  | 9,975 | 67 | 6.7 |  | 9,878 | 63 | 6.4 |  |
| Lebanon | 2003 |  | 6,999 | 41 | 5.9 |  | 6,963 | 44 | 6.3 |  | 6,795 | 41 | 6 |  |
| Lebanon | 2004 |  | 9,400 | 66 | 7 |  | 9,180 | 62 | 6.8 |  | 9,019 | 56 | 6.2 |  |
| Lebanon | 2005 |  | 13,246 | 89 | 6.7 |  | 13,126 | 80 | 6.1 |  | 12,608 | 70 | 5.6 |  |
| Lebanon | 2006 |  | 14,584 | 113 | 7.7 |  | 14,484 | 107 | 7.4 |  | 14,073 | 98 | 7 |  |
| Lebanon | 2007 |  | 15,538 | 109 | 7 |  | 15,592 | 110 | 7.1 |  | 14,874 | 88 | 5.9 |  |
| Lebanon | 2008 |  | 17,558 | 88 | 5 |  | 17,447 | 95 | 5.4 |  | 16,839 | 83 | 4.9 |  |
| Lebanon | 2009 |  | 19,776 | 82 | 4.1 |  | 19,067 | 80 | 4.2 |  | 18,602 | 74 | 4 |  |
| Lebanon | 2010 |  | 21,126 | 83 | 3.9 |  | 20,813 | 78 | 3.7 |  | 20,141 | 71 | 3.5 |  |
| Lebanon | 2011 |  | 21,902 | 70 | 3.2 |  | 21,595 | 70 | 3.2 |  | 20,775 | 64 | 3.1 |  |
| Lebanon | 2012 |  | 25,028 | 68 | 2.7 |  | 24,766 | 70 | 2.8 |  | 23,853 | 61 | 2.6 |  |
| Lebanon | 2013 |  | 26,191 | 52 | 2 |  | 25,930 | 51 | 2 |  | 25,030 | 50 | 2 |  |
| Lebanon | 2014 |  | 21,211 | 60 | 2.8 |  | 21,039 | 56 | 2.7 |  | 20,533 | 53 | 2.6 |  |
| Lebanon | 2015 |  | 17,823 | 47 | 2.6 |  | 17,782 | 46 | 2.6 |  | 17,227 | 45 | 2.6 |  |
| Lebanon | 2016 |  | 17,576 | 37 | 2.1 |  | 17,541 | 34 | 1.9 |  | 16,918 | 33 | 2 |  |
| Lebanon | 2017 | 5.5 | 16,248 | 43 | 2.6 | -2.9 | 16,240 | 43 | 2.6 | -2.9 | 15,815 | 42 | 2.7 | -2.8 |
| Lebanon | 2018 |  | 16,506 | 30 | 1.8 |  | 16,586 | 29 | 1.7 |  | 16,173 | 28 | 1.7 |  |
| Lebanon | 2019 |  | 13,793 | 54 | 3.9 |  | 13,809 | 55 | 4 |  | 13,461 | 52 | 3.9 |  |
| Mexico | 2017 | 7.2 | 1,948,078 | 11,992 | 6.2 | -1 | 1,948,078 | 11,992 | 6.2 | -1 | 1,948,078 | 11,992 | 6.2 | -1 |
| Mexico | 2018 | 6.7 | 1,848,862 | 11,279 | 6.1 | -0.6 | 1,848,862 | 11,279 | 6.1 | -0.6 | 1,848,862 | 11,279 | 6.1 | -0.6 |
| Mexico | 2019 | 6.5 | 1,763,976 | 10,765 | 6.1 | -0.4 | 1,763,976 | 10,765 | 6.1 | -0.4 | 1,763,976 | 10,765 | 6.1 | -0.4 |
| Netherlands | 2010 | 2.8 | 177,512 | 712 | 4 | 1.2 | 176,457 | 586 | 3.3 | 0.5 | 176,296 | 582 | 3.3 | 0.5 |
| Netherlands | 2011 | 2.7 | 175,582 | 709 | 4 | 1.3 | 174,521 | 568 | 3.3 | 0.6 | 174,300 | 564 | 3.2 | 0.5 |
| Netherlands | 2012 | 2.6 | 173,551 | 637 | 3.7 | 1.1 | 171,725 | 510 | 3 | 0.4 | 171,497 | 507 | 3 | 0.4 |
| Netherlands | 2013 | 2.8 | 168,186 | 663 | 3.9 | 1.1 | 167,046 | 527 | 3.2 | 0.4 | 166,833 | 525 | 3.1 | 0.3 |
| Netherlands | 2014 | 2.7 | 172,170 | 653 | 3.8 | 1.1 | 170,717 | 502 | 2.9 | 0.2 | 170,486 | 496 | 2.9 | 0.2 |
| Netherlands | 2015 | 2.5 | 166,913 | 587 | 3.5 | 1 | 165,793 | 477 | 2.9 | 0.4 | 165,525 | 470 | 2.8 | 0.3 |
| Netherlands | 2016 | 2.6 | 169,682 | 623 | 3.7 | 1.1 | 168,769 | 496 | 2.9 | 0.3 | 168,507 | 490 | 2.9 | 0.3 |
| Netherlands | 2017 | 2.7 | 166,513 | 661 | 4 | 1.3 | 165,468 | 536 | 3.2 | 0.5 | 165,147 | 528 | 3.2 | 0.5 |
| Netherlands | 2018 | 2.6 | 162,423 | 645 | 4 | 1.4 | 161,636 | 534 | 3.3 | 0.7 | 161,157 | 520 | 3.2 | 0.6 |
| Netherlands | 2019 | 2.7 | 164,565 | 665 | 4 | 1.3 | 163,533 | 510 | 3.1 | 0.4 | 163,263 | 496 | 3 | 0.3 |
| Northern Ireland | 2016-2021 | 2.7 | 155,992 | 290 | 1.9 | -0.8 | 155,906 | 290 | 1.9 | -0.8 | 155,992 | 290 | 1.9 | -0.8 |
| Qatar | 2016 | 3.3 | 22,035 | 70 | 3.2 | -0.1 | 22,035 | 70 | 3.2 | -0.1 | 22,035 | 70 | 3.2 | -0.1 |
| Qatar | 2017 | 3.8 | 23,936 | 88 | 3.7 | -0.1 | 23,936 | 88 | 3.7 | -0.1 | 23,936 | 88 | 3.7 | -0.1 |
| Qatar | 2018 | 3.8 | 23,549 | 67 | 2.8 | -1 | 23,549 | 67 | 2.8 | -1 | 23,549 | 67 | 2.8 | -1 |
| Qatar | 2019 | 3.7 | 24,817 | 72 | 2.9 | -0.8 | 24,817 | 72 | 2.9 | -0.8 | 24,817 | 72 | 2.9 | -0.8 |
| Scotland | 2000 | 3.9 | 52,492 | 189 | 3.6 | -0.3 | 52,495 | 191 | 3.6 | -0.3 | 52,474 | 188 | 3.6 | -0.3 |
| Scotland | 2001 | 3.7 | 51,279 | 175 | 3.4 | -0.3 | 51,269 | 171 | 3.3 | -0.4 | 51,255 | 169 | 3.3 | -0.4 |
| Scotland | 2002 | 3.6 | 50,442 | 134 | 2.7 | -0.9 | 50,436 | 136 | 2.7 | -0.9 | 50,418 | 133 | 2.6 | -1 |
| Scotland | 2003 | 3.6 | 51,557 | 156 | 3 | -0.6 | 51,560 | 156 | 3 | -0.6 | 51,531 | 154 | 3 | -0.6 |
| Scotland | 2004 | 3.5 | 53,098 | 144 | 2.7 | -0.8 | 53,090 | 144 | 2.7 | -0.8 | 53,070 | 140 | 2.6 | -0.9 |
| Scotland | 2005 | 3.5 | 52,794 | 150 | 2.8 | -0.7 | 52,818 | 150 | 2.8 | -0.7 | 52,769 | 148 | 2.8 | -0.7 |
| Scotland | 2006 | 3.5 | 53,473 | 136 | 2.5 | -1 | 53,476 | 129 | 2.4 | -1.1 | 53,433 | 127 | 2.4 | -1.1 |
| Scotland | 2007 | 3.3 | 56465 | 153 | 2.7 | -0.6 | 56,493 | 145 | 2.6 | -0.7 | 56,418 | 141 | 2.5 | -0.8 |
| Scotland | 2008 | 3.2 | 58,616 | 144 | 2.5 | -0.7 | 58,621 | 136 | 2.3 | -0.9 | 58,553 | 133 | 2.3 | -0.9 |
| Scotland | 2009 | 3.2 | 57812 | 143 | 2.5 | -0.7 | 57,823 | 136 | 2.4 | -0.8 | 57,759 | 133 | 2.3 | -0.9 |
| Scotland | 2010 | 3 | 57,840 | 123 | 2.1 | -0.9 | 57,826 | 121 | 2.1 | -0.9 | 57,807 | 120 | 2.1 | -0.9 |
| Scotland | 2011 | 3 | 57,492 | 138 | 2.4 | -0.6 | 57,463 | 135 | 2.3 | -0.7 | 57,455 | 135 | 2.3 | -0.7 |
| Scotland | 2012 | 2.9 | 56,831 | 132 | 2.3 | -0.6 | 56,804 | 124 | 2.2 | -0.7 | 56,774 | 123 | 2.2 | -0.7 |
| Scotland | 2013 | 2.7 | 54,961 | 113 | 2.1 | -0.6 | 54,983 | 112 | 2 | -0.7 | 54,886 | 109 | 2 | -0.7 |
| Scotland | 2014 | 2.7 | 55,817 | 128 | 2.3 | -0.4 | 55,653 | 121 | 2.2 | -0.5 | 55,578 | 121 | 2.2 | -0.5 |
| Scotland | 2015 | 2.7 | 54,352 | 90 | 1.7 | -1 | 54,169 | 83 | 1.5 | -1.2 | 54,111 | 83 | 1.5 | -1.2 |
| Scotland | 2016 | 2.8 | 53684 | 112 | 2.1 | -0.7 | 53,371 | 104 | 1.9 | -0.9 | 53,256 | 101 | 1.9 | -0.9 |
| Scotland | 2017 | 2.8 | 51,930 | 104 | 2 | -0.8 | 51,945 | 100 | 1.9 | -0.9 | 51,659 | 98 | 1.9 | -0.9 |
| Scotland | 2018 | 2.8 | 50,374 | 88 | 1.7 | -1.1 | 50,530 | 85 | 1.7 | -1.1 | 50,333 | 82 | 1.6 | -1.2 |
| Scotland | 2019 | 2.8 | 48,637 | 87 | 1.8 | -1 | 48,677 | 84 | 1.7 | -1.1 | 48,622 | 81 | 1.7 | -1.1 |
| Scotland | 2020 | 2.8 | 46,678 | 83 | 1.8 | -1 | 46,697 | 80 | 1.7 | -1.1 | 46,656 | 80 | 1.7 | -1.1 |
| Sweden | 2000-2019 | 1.4 | 2,102,671 | 2,758 | 1.3 | -0.1 | 2,102,671 | 2,755 | 1.3 | -0.1 | 2,102,671 | 2,755 | 1.3 | -0.1 |
| England & Wales | 2015 | 2.7 | 666,881 | 1,527 | 2.3 | -0.4 | 666,881 | 1,527 | 2.3 | -0.4 | 666,881 | 1,527 | 2.3 | -0.4 |
| England & Wales | 2016 | 2.8 | 666,539 | 1,490 | 2.2 | -0.6 | 666,539 | 1,490 | 2.2 | -0.6 | 666,539 | 1,490 | 2.2 | -0.6 |
| England & Wales | 2017 | 2.8 | 649,066 | 1,454 | 2.2 | -0.6 | 649,066 | 1,454 | 2.2 | -0.6 | 649,066 | 1,454 | 2.2 | -0.6 |
| England & Wales | 2018 | 2.8 | 621,468 | 1,385 | 2.2 | -0.6 | 621,468 | 1,385 | 2.2 | -0.6 | 621,468 | 1,385 | 2.2 | -0.6 |
| England & Wales | 2019 | 2.8 | 608,538 | 1,313 | 2.2 | -0.6 | 608,538 | 1,313 | 2.2 | -0.6 | 608,538 | 1,313 | 2.2 | -0.6 |
| Uruguay | 2009 | 4.3 | 41,104 | 35 | 0.9 | -3.4 | 40,090 | 35 | 0.9 | -3.4 | 39,970 | 35 | 0.9 | -3.4 |
| Uruguay | 2010 | 3.9 | 33,771 | 23 | 0.7 | -3.2 | 33,247 | 23 | 0.7 | -3.2 | 33,162 | 23 | 0.7 | -3.2 |
| Uruguay | 2011 | 3.9 | 41,866 | 91 | 2.2 | -1.7 | 41,277 | 89 | 2.2 | -1.7 | 41,210 | 87 | 2.1 | -1.8 |
| Uruguay | 2012 | 5.6 | 45,326 | 69 | 1.5 | -4.1 | 44,846 | 69 | 1.5 | -4.1 | 44,758 | 68 | 1.5 | -4.1 |
| Uruguay | 2013 | 5.3 | 46,369 | 49 | 1.1 | -4.2 | 45,998 | 49 | 1.1 | -4.2 | 45,925 | 48 | 1 | -4.3 |
| Uruguay | 2014 | 5 | 46,825 | 46 | 1 | -4 | 46,506 | 46 | 1 | -4 | 46,456 | 46 | 1 | -4 |
| Uruguay | 2015 | 5 | 46,842 | 50 | 1.1 | -3.9 | 46,534 | 51 | 1.1 | -3.9 | 46,477 | 50 | 1.1 | -3.9 |
| Uruguay | 2016 | 5.2 | 45,459 | 78 | 1.7 | -3.5 | 45,156 | 78 | 1.7 | -3.5 | 45,103 | 77 | 1.7 | -3.5 |
| Uruguay | 2017 | 4.3 | 41,884 | 50 | 1.2 | -3.1 | 41,664 | 50 | 1.2 | -3.1 | 41,619 | 50 | 1.2 | -3.1 |
| Uruguay | 2018 | 3.9 | 38,771 | 48 | 1.2 | -2.7 | 38,503 | 48 | 1.2 | -2.7 | 38,466 | 48 | 1.2 | -2.7 |
| Uruguay | 2019 | 3.5 | 35,662 | 68 | 1.9 | -1.6 | 35,387 | 68 | 1.9 | -1.6 | 35,343 | 68 | 1.9 | -1.6 |
| Uruguay | 2020 | 4.3 | 34,886 | 65 | 1.9 | -2.4 | 34,581 | 64 | 1.9 | -2.4 | 34,551 | 64 | 1.9 | -2.4 |
| USA | 2000 | 4.6 | 4,061,092 | 18,120 | 4.5 | -0.1 | 4,011,706 | 14,336 | 3.6 | -1 | 4,011,112 | 14,253 | 3.6 | -1 |
| USA | 2001 | 4.5 | 4,030,307 | 17,810 | 4.4 | -0.1 | 3,982,855 | 13,947 | 3.5 | -1 | 3,982,387 | 13,861 | 3.5 | -1 |
| USA | 2002 | 4.7 | 4,026,197 | 18,136 | 4.5 | -0.2 | 3,977,311 | 14,174 | 3.6 | -1.1 | 3,976,859 | 14,107 | 3.5 | -1.2 |
| USA | 2003 | 4.6 | 4,095,477 | 18,572 | 4.5 | -0.1 | 4,043,012 | 14,215 | 3.5 | -1.1 | 4,042,904 | 14,197 | 3.5 | -1.1 |
| USA | 2004 | 4.5 | 4,118,277 | 18,323 | 4.4 | -0.1 | 4,066,556 | 14,037 | 3.5 | -1 | 4,066,505 | 14,022 | 3.4 | -1.1 |
| USA | 2005 | 4.5 | 4,145,258 | 18,435 | 4.4 | -0.1 | 4,107,177 | 14,075 | 3.4 | -1.1 | 4,107,089 | 14,044 | 3.4 | -1.1 |
| USA | 2006 | 4.5 | 4,272,106 | 18,640 | 4.4 | -0.1 | 4,238,782 | 14,410 | 3.4 | -1.1 | 4,238,731 | 14,392 | 3.4 | -1.1 |
| USA | 2007 | 4.4 | 4,323,391 | 18,626 | 4.3 | -0.1 | 4,311,757 | 14,359 | 3.3 | -1.1 | 4,311,708 | 14,341 | 3.3 | -1.1 |
| USA | 2008 | 4.3 | 4,254,111 | 17,859 | 4.2 | -0.1 | 4,244,010 | 13,573 | 3.2 | -1.1 | 4,243,964 | 13,553 | 3.2 | -1.1 |
| USA | 2009 | 4.2 | 4,136,809 | 16,899 | 4.1 | -0.1 | 4,127,677 | 12,907 | 3.1 | -1.1 | 4,127,631 | 12,897 | 3.1 | -1.1 |
| USA | 2010 | 4.1 | 4,006,465 | 15,892 | 4 | -0.1 | 3,997,225 | 12,205 | 3.1 | -1 | 3,997,192 | 12,191 | 3 | -1.1 |
| USA | 2011 | 4.1 | 3,959,481 | 15,709 | 4 | -0.1 | 3,951,654 | 11,942 | 3 | -1.1 | 3,951,607 | 11,934 | 3 | -1.1 |
| USA | 2012 | 4 | 3,960,052 | 15,615 | 3.9 | -0.1 | 3,952,011 | 11,721 | 3 | -1 | 3,951,961 | 11,700 | 3 | -1 |
| USA | 2013 | 4 | 3,940,035 | 15,576 | 4 | 0 | 3,929,746 | 11,482 | 2.9 | -1.1 | 3,929,692 | 11,461 | 2.9 | -1.1 |
| USA | 2014 | 3.9 | 3,997,358 | 15,498 | 3.9 | 0 | 3,990,370 | 11,552 | 2.9 | -1 | 3,990,322 | 11,539 | 2.9 | -1 |
| USA | 2015 | 3.9 | 3,987,951 | 15,440 | 3.9 | 0 | 3,981,482 | 11,604 | 2.9 | -1 | 3,981,428 | 11,581 | 2.9 | -1 |
| USA | 2016 | 3.9 | 3,942,236 | 15,046 | 3.8 | -0.1 | 3,938,207 | 11,271 | 2.9 | -1 | 3,935,732 | 11,259 | 2.9 | -1 |
| USA | 2017 | 3.8 | 3,855,948 | 14,622 | 3.8 | 0 | 3,851,269 | 10,891 | 2.8 | -1 | 3,850,067 | 10,874 | 2.8 | -1 |
| USA | 2018 | 3.7 | 3,792,248 | 14,069 | 3.7 | 0 | 3,787,911 | 10,513 | 2.8 | -0.9 | 3,786,657 | 10,496 | 2.8 | -0.9 |
| USA | 2019 | 3.5 | 3,747,254 | 13,560 | 3.6 | 0.1 | 3,743,787 | 10,061 | 2.7 | -0.8 | 3,741,978 | 10,043 | 2.7 | -0.8 |

Canada: excluding data from Quebec and mortality after discharge

# **Table S5. Number of missing values on three core variables (birthweight, gestational age, and sex) among included country-years**

| **Country** | **Period of observation** | **Livebirths** | **Missing values** | | | | | |
| --- | --- | --- | --- | --- | --- | --- | --- | --- |
|  |  |  | **Birthweight** | | **Gestational age** | | **Sex** | |
|  | **(years)** | **(n)** | **(n)** | **(%)** | **(n)** | **(%)** | **(n)** | **(%)** |
| Australia | 2016-2019 | 1,217,919 | 539 | <0.1 | 548 | <0.1 | 108 | <0.1 |
| Brazil | 2011-2018 | 23,439,789 | 13,442 | 0.1 | 2,036,695 | 8.7 | 4,217 | <0.1 |
| Canada | 2005-2020 | 4,163,541 | 347 | <0.1 | 34,794 | 0.8 | 160 | <0.1 |
| Czech Republic | 2019 | 112,231 | 1,512 | 1.3 | 2,719 | 2.4 | 0 | 0.0 |
| Denmark | 2000-2017 | 1,125,560 | 20,903 | 1.9 | 18,748 | 1.7 | 294 | <0.1 |
| England & Wales | 2015-2019 | 3,212,492 | 0 | 0.0 | 0 | 0.0 | 0 | 0.0 |
| Estonia | 2015-2020 | 82,427 | 0 | 0.0 | 0 | 0.0 | 0 | 0.0 |
| Lebanon | 2001 & 2017 | 26,792 | 535 | 2.0 | 709 | 2.6 | 677 | 2.5 |
| Netherlands | 2010-2019 | 1,861,400 | 2,277 | 0.1 | 12,520 | 0.7 | 59 | <0.1 |
| Qatar | 2016-2019 | 95,906 | 350 | 0.4 | 1,202 | 1.3 | 5 | <0.1 |
| Scotland | 2000-2020 | 1,127,984 | 1,357 | 0.1 | 1,659 | 0.1 | 181 | <0.1 |
| Sweden | 2000-2019 | 2,102,671 | 0 | 0.0 | 0 | 0.0 | 0 | 0.0 |
| Uruguay | 2009-2020 | 499,345 | 580 | 0.1 | 5,350 | 1.1 | 0 | 0.0 |
| US | 2000-2019 | 80,710,348 | 78,490 | 0.1 | 317,158 | 0.4 | 1,672 | <0.1 |

# **Table S6. Summary of metadata**

| **Country** | **Units for reporting**  **Birthweight/ Gestational age** | **Data linkage** | **Reporting criteria for very preterm** | | |
| --- | --- | --- | --- | --- | --- |
|  |  |  | **Exclusions criteria based on BW** | **Exclusion criteria based on GA** | **Are births following induced Termination of Pregnancy included in the data source?** |
| Australia | Grams | Not applicable as livebirths, stillbirths and neonatal deaths are all included as part of the National Perinatal Data Collection | A small number of births <400 grams are included | A small number of births < 20 weeks are included | Both livebirths and stillbirths may include termination of pregnancy after 20 weeks. |
|  | Completed weeks |  |  |  |  |
| Brazil | Grams | Livebirths and death records were linked using the name of the mother, maternal date of birth or age (when the date of birth was missing), and the municipality of residence of the mother as matching variables using CIDACS-RL software | <350g | <20 weeks | No |
|  | Completed weeks |  |  |  |  |
| Canada | Grams | Data were obtained from the Discharge Abstract Database (DAD) which collates all hospitalization records of maternal hospitalizations for childbirth and also links maternal and live birth infant records. The province of Quebec does not contribute data to DAD. Home births were not included | None | None | No |
|  | Completed weeks |  |  |  |  |
| Czech Republic | Grams | Information system of newborn is linked at the individual level to the Registry of Reproductive Health and Information system of the dead | None | <22 weeks | No |
|  | Weeks +days |  |  |  |  |
| Denmark | Grams | Information on livebirths and stillbirths were extracted from the Danish Medical Birth Registry. These data were linked to the Danish Civil Registration System to define infant death | None | None | No |
|  | Days |  |  |  |  |
| England & Wales | Grams | Data linkage with birth notifications, and birth and stillbirth registrations and neonatal death registrations | None | 22 weeks | No |
|  | Completed weeks |  |  |  |  |
| Estonia | Grams | Estonian Medical Birth Register is Linked at the individual level to the Registry of Causes of Death | None | <22 weeks | No |
|  | Weeks +days |  |  |  |  |
| Lebanon | Grams  Weeks+ days |  | None | <22 weeks | No |
| Mexico | Weeks +days | Livebirths and deaths records were linked using the variables sex, date of birth, place of residence and place of occurrence using CIDACS-RL software | None | None | No |
|  | Completed weeks |  |  |  |  |
| Netherlands | Grams  Weeks + days | Records from midwifes, clinical obstetricians/gynaecologists and neonatologist are linked nationally | Gestational age ≥22 weeks; if g.a. missing, birthweight ≥500 g | Gestational age ≥22 weeks; if g.a. missing, birthweight ≥500 g | Both livebirths and stillbirths up to 24 weeks gestation may include termination of pregnancy |
| Qatar | Kilograms and grams  Weeks and days | Not applicable | None | None | No |
| Scotland | Grams | SMR02 is a file based on babies (live and stillbirths) was matched with NHS Lothian file and NRS infant deaths | None | None | No induced ToPs are included |
|  | Completed weeks |  |  |  |  |
| Sweden | Grams | National databases that are linked together using the person-unique national registration numbers of children | None | <23 weeks | Included after 22 weeks |
|  | Days |  |  |  |  |
| Uruguay | Grams |  | None | None | No |
|  | Completed weeks |  |  |  |  |
| United States of America | Grams Completed weeks | Data on live births are available from the NCHS – Vital Statistics online. It includes data from live birth certificates. Linkages are performed to obtain information on infant death from Vital Statistics; the proportion of linked infant deaths is generally high, it differs by year (overall 98.7%-99.4%), and by state (e.g., 92.8%-100% in 2000) | None | None | The NCHS recommendation for fetal death definition is to exclude TOPs. Some states do exclude TOPs regardless of gestational age, while some states include TOPs |

# **Figure S1. Neonatal mortality NMRs by gestational age among very preterm births (22 to 32 completed weeks) in 15 countries**

NMR: number of neonatal deaths / number of livebirths per 1000


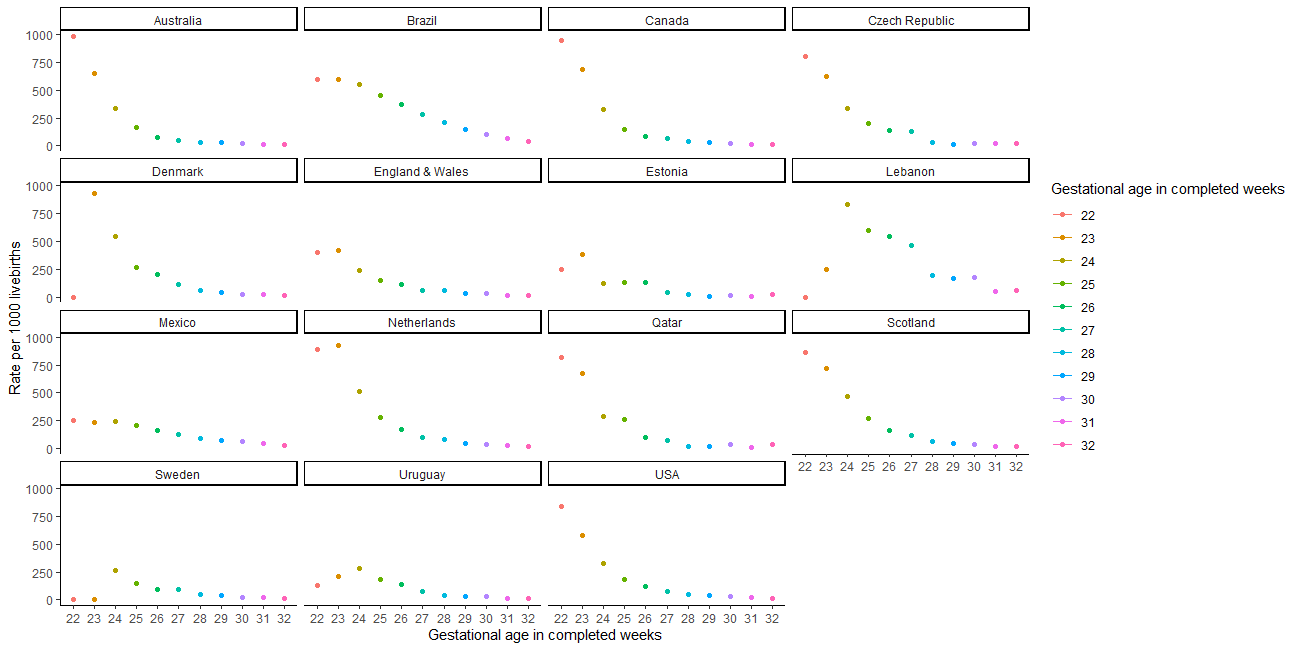


Each point indicates the NMR for each gestational age in completed weeks

# **Figure S2. Neonatal mortality NMRs by gestational age among very preterm births (22 to 32 completed weeks) by region**


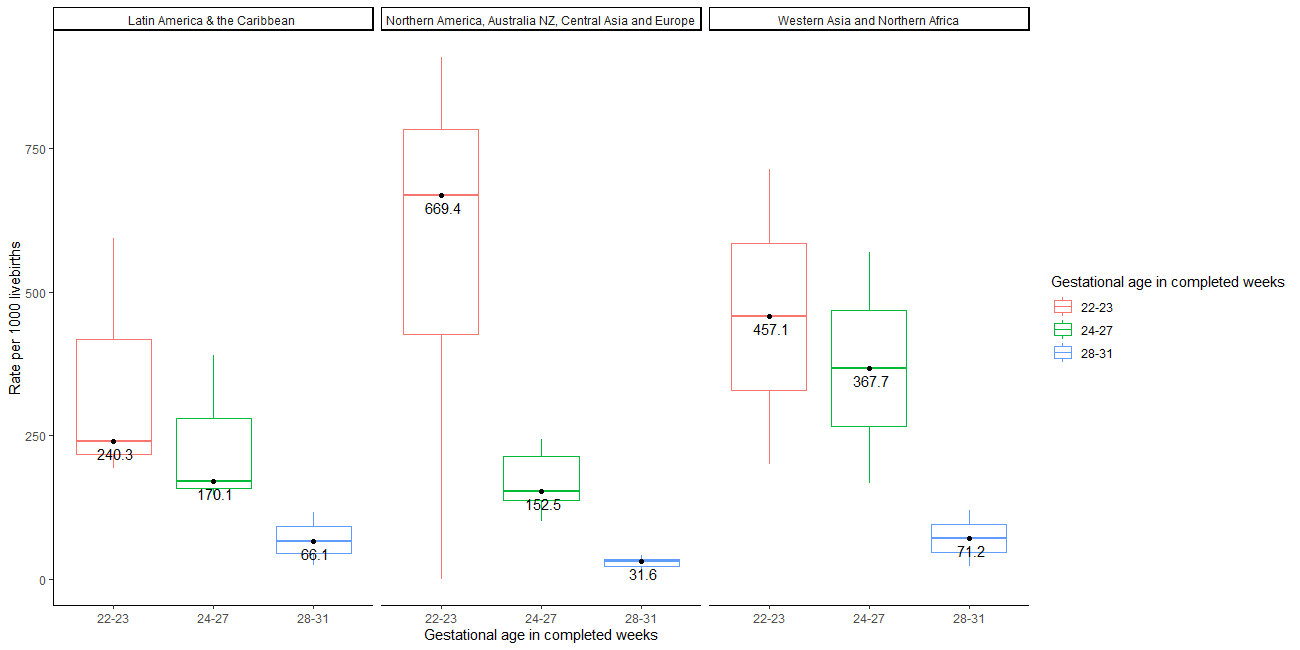


Numbers show median NMRs and box plots summarize the median, 25^th^ and 75^th^ percentiles

# **Table S7. Ranking of neonatal mortality NMRs comparing two groups of babies (≥22 weeks and ≥24 weeks) in 15 countries**

| **Ranking** | **≥22 weeks** | | | **Ranking** | **≥24 weeks** | | | |
| --- | --- | --- | --- | --- | --- | --- | --- | --- |
|  | **Livebirths** | **Deaths** | **NMR** |  | **Livebirths** | **Deaths** | | **NMR** |
|  | **(n)** | **(n)** | **per 1000** |  | **(n)** | **(n)** | | **per 1000** |
| Brazil | 21,355,008 | 149,234 | 7.0 | Brazil | 21,334,724 | 137,181 | | 6.4 |
| Mexico | 5,560,916 | 34,036 | 6.1 | Mexico | 5,559,909 | 33,794 | | 6.1 |
| Lebanon | 25,966 | 123 | 4.7 | Lebanon | 25,961 | 122 | | 4.7 |
| USA | 80,234,505 | 253,275 | 3.2 | Qatar | 94,239 | 227 | | 2.4 |
| Qatar | 94,337 | 297 | 3.1 | USA | 80,132,448 | 182,680 | | 2.3 |
| Netherlands | 1,849,432 | 5,556 | 3.0 | Denmark | 1,100,286 | 2,324 | | 2.1 |
| Denmark | 1,100,854 | 2,598 | 2.4 | Netherlands | 1,847,584 | 3,875 | | 2.1 |
| Scotland | 1,126,199 | 2,643 | 2.3 | Scotland | 1,125,732 | 2,288 | | 2.0 |
| England and Wales | 3,212,492 | 7,169 | 2.2 | England and Wales | 3,208,542 | 5,560 | | 1.7 |
| Australia | 1,216,690 | 2,166 | 1.8 | Czech Republic | 109,458 | 146 | | 1.3 |
| Canada | 4,386,246 | 7,794 | 1.8 | Sweden | 2,102,671 | 2,755 | | 1.3 |
| Czech Republic | 109,492 | 168 | 1.5 | Uruguay | 493,613 | 636 | | 1.3 |
| Uruguay | 493,789 | 670 | 1.4 | Australia | 1,215,746 | 1,419 | | 1.2 |
| Sweden | 2,102,671 | 2,755 | 1.3 | Canada | 4,382,662 | 4,935 | | 1.1 |
| Estonia | 82,427 | 95 | 1.2 | Estonia | 82,384 | 80 | | 1.0 |
| **Region key** | | | | | | |  |  |
| Latin America and the Caribbean | | | | | | |  |  |
| Northern America, Australia and New Zealand, Central Asia and Europe | | | | | | |  |  |
| Western Asia and Northern Africa | | | | | | |  |  |

# **Figure S3. Overview of Vulnerable newborn types based on gestational age, size for gestational age and birthweight.**

1. Six newborn types used in the main analysis.


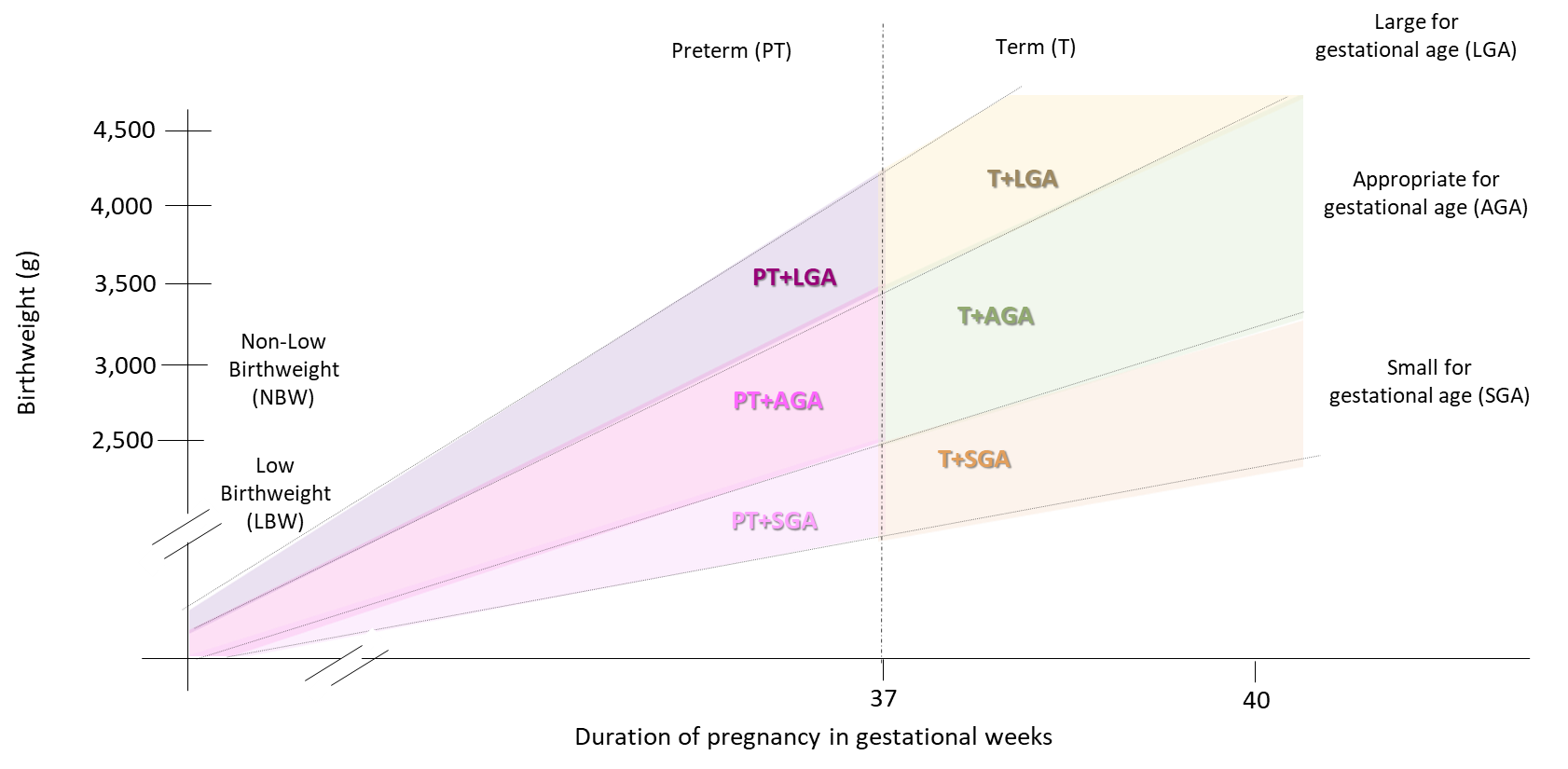


1. Ten newborn types used in the secondary analysis - including birthweight dimension


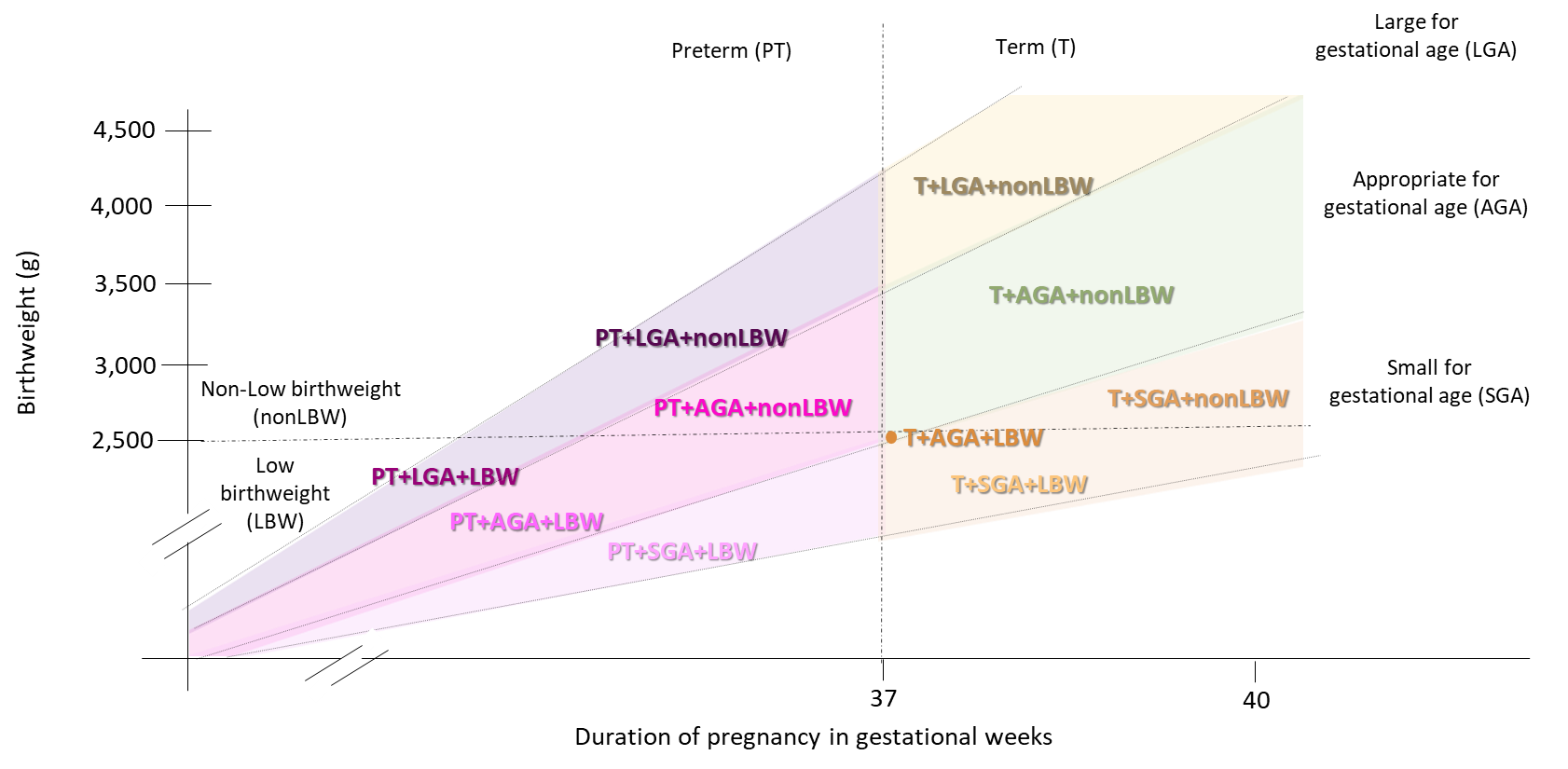


1. Original newborn types proposed by Ashorn *et al ^7^*


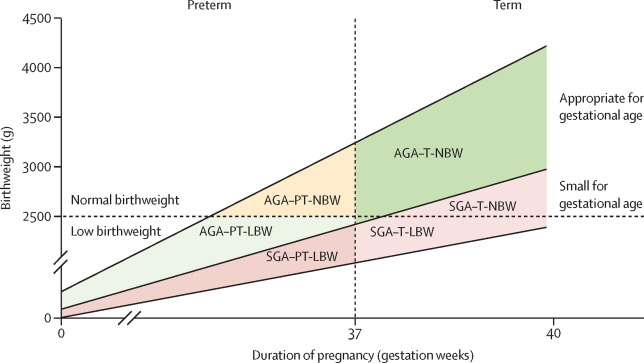


This figure illustrates the six newborn types (a) used in the main analysis and more granular expansion of these types adding the birthweight dimension (b) used in the secondary analysis in this paper.

# **Additional results:**

# **Table S8. Neonatal mortality NMR, population attributable risk, and crude relative risk of neonatal mortality for birthweight fine strata (reference 2,500g to 4,000g), by country**

Prevalence = the number of livebirths reported in each group of interest / total number of livebirths

Neonatal Mortality NMR (NMR): the number of persons who experienced the event (neonatal death) divided by the total number of persons exposed to the risk of that event per 1000

Population attributable risk (PAR): was calculated for each level of exposure with the following formula where *pr* is the prevalence and *RR* is the relative risk ^8^

$PAR for type of interest= \frac{pr\left( type of interest \right)\left( RR\left( type of interest-1 \right) \right)}{\sum_{all types} pr\left( type \right)x RR(type)}$

Relative risk: the absolute risk within a specific category divided by the absolute risk in the reference group, expressed as a ratio

| **Country** | **Total** | | | **2,500g to 4,000g** | | | |
| --- | --- | --- | --- | --- | --- | --- | --- |
|  | **Livebirths** | **Deaths** | **Rate** | **Livebirths** | **Deaths** | **Prevalence** | **Rate** |
|  | **(n)** | **(n)** | **per 1000** | **(n)** | **(n)** | **(%)** | **per 1000** |
| Australia | 1,217,328 | 2,734 | 2 | 1,020,141 | 436 | 83.8 | 0.4 |
| Brazil | 23,426,090 | 173,155 | 7.4 | 20,247,168 | 45,452 | 86.4 | 2.2 |
| Canada | 4,429,455 | 10,177 | 2.3 | 3,655,142 | 1,144 | 82.5 | 0.3 |
| Czech Republic | 112,231 | 174 | 1.6 | 94,014 | 42 | 83.8 | 0.4 |
| Denmark | 1,100,854 | 2,598 | 2.4 | 848,717 | 654 | 77.1 | 0.8 |
| England and Wales | 3,212,492 | 7,169 | 2.2 | 2,636,657 | 1,844 | 82.1 | 0.7 |
| Estonia | 82,310 | 95 | 1.2 | 64,153 | 27 | 77.9 | 0.4 |
| Lebanon | 26,257 | 118 | 4.5 | 22,859 | 40 | 87.1 | 1.7 |
| Mexico | 5,560,916 | 34,036 | 6.1 | 5,039,848 | 25,819 | 90.6 | 5.1 |
| Netherlands | 1,861,306 | 6,866 | 3.7 | 1,498,550 | 1,220 | 80.5 | 0.8 |
| Qatar | 94,337 | 297 | 3.1 | 81,009 | 55 | 85.9 | 0.7 |
| Scotland | 1,126,627 | 2,722 | 2.4 | 900,470 | 698 | 79.9 | 0.8 |
| Sweden | 2,102,671 | 2,758 | 1.3 | 1,626,048 | 964 | 77.3 | 0.6 |
| Uruguay | 498,765 | 672 | 1.3 | 421,206 | 153 | 84.4 | 0.4 |
| USA | 80,652,053 | 332,447 | 4.1 | 67,554,421 | 51,529 | 83.8 | 0.8 |

| **Country** | **<1,000g** | | | | | | |
| --- | --- | --- | --- | --- | --- | --- | --- |
|  | **Livebirths** | **Deaths** | **Prevalence** | **Rate** | **PAR** | **Relative risk** | **95%CI** |
|  | **(n)** | **(n)** | **(%)** | **per 1000** | **(%)** | **(n)** | **(n)** |
| Australia | 5,178 | 1,768 | 0.4 | 341.4 | 57.8 | 595.8 | (537.9, 659.9) |
| Brazil | 142,717 | 64,898 | 0.6 | 454.7 | 29.8 | 139.6 | (138.0, 141.1) |
| Canada | 19,957 | 7,063 | 0.5 | 353.9 | 62.7 | 835.4 | (785.8, 888.3) |
| Czech Republic | 1,895 | 84 | 1.7 | 44.3 | 46.9 | 95.1 | (65.8, 137.3) |
| Denmark | 3,605 | 1,255 | 0.3 | 348.1 | 41.1 | 335.4 | (306.4, 367.0) |
| England and Wales | 17,568 | 3,273 | 0.5 | 186.3 | 41.5 | 224.7 | (212.6, 237.5) |
| Estonia | 261 | 31 | 0.3 | 118.8 | 30.3 | 252.4 | (152.6, 417.3) |
| Lebanon | 77 | 39 | 0.3 | 506.5 | 25.4 | 192.5 | (128.8, 287.6) |
| Mexico | 13,409 | 2,172 | 0.2 | 162.0 | 5.4 | 27.4 | (26.3, 28.5) |
| Netherlands | 9,635 | 4,309 | 0.5 | 447.2 | 53.9 | 379.9 | (357.3, 403.9) |
| Qatar | 489 | 140 | 0.5 | 286.3 | 41.2 | 328.1 | (242.6, 443.7) |
| Scotland | 4,296 | 1,205 | 0.4 | 280.5 | 38.4 | 282.8 | (258.6, 309.3) |
| Sweden | 5,261 | 695 | 0.3 | 132.1 | 23.0 | 196.9 | (179.2, 216.4) |
| Uruguay | 2,237 | 306 | 0.4 | 136.8 | 42.4 | 331.4 | (274.0, 400.8) |
| USA | 579,635 | 211,113 | 0.7 | 364.2 | 56.2 | 350.3 | (347.0, 353.6) |

| Country | **1,000g to 1,500g** | | | | | | |
| --- | --- | --- | --- | --- | --- | --- | --- |
|  | **Livebirths** | **Deaths** | **Prevalence** | **Rate** | **PAR** | **Relative risk** | **95%CI** |
|  | **(n)** | **(n)** | **(%)** | **per 1000** | **(%)** | **(n)** | **(n)** |
| Australia | 6,620 | 187 | 0.5 | 28.2 | 7.9 | 64.3 | (54.3, 76.2) |
| Brazil | 174,547 | 25,529 | 0.7 | 146.3 | 14.7 | 57.0 | (56.1, 57.8) |
| Canada | 25,040 | 709 | 0.6 | 28.3 | 8.2 | 88.0 | (80.2, 96.6) |
| Czech Republic | 669 | 21 | 0.6 | 31.4 | 11.8 | 68.2 | (40.6, 114.5) |
| Denmark | 5,835 | 258 | 0.5 | 44.2 | 10.7 | 55.0 | (47.7, 63.4) |
| England and Wales | 19,500 | 666 | 0.6 | 34.2 | 9.5 | 47.3 | (43.3, 51.6) |
| Estonia | 362 | 14 | 0.4 | 38.7 | 14.6 | 88.5 | (46.7, 167.4) |
| Lebanon | 155 | 24 | 0.6 | 154.8 | 20.2 | 76.8 | (47.3, 124.6) |
| Mexico | 25,170 | 1,599 | 0.5 | 63.5 | 4.1 | 11.7 | (11.2, 12.3) |
| Netherlands | 10,538 | 466 | 0.6 | 44.2 | 7.9 | 52.1 | (46.9, 57.8) |
| Qatar | 738 | 33 | 0.8 | 44.7 | 11.8 | 63.1 | (41.2, 96.6) |
| Scotland | 7,594 | 305 | 0.7 | 40.2 | 11.8 | 49.9 | (43.7, 56.9) |
| Sweden | 9,066 | 338 | 0.4 | 37.3 | 12.1 | 60.7 | (53.7, 68.5) |
| Uruguay | 3,809 | 90 | 0.8 | 23.6 | 13.7 | 63.6 | (49.1, 82.3) |
| USA | 599,506 | 22,967 | 0.7 | 38.3 | 7.9 | 48.4 | (47.7, 49.2) |

| Country | **1,500g to 2,000g** | | | | | | |
| --- | --- | --- | --- | --- | --- | --- | --- |
|  | **Livebirths** | **Deaths** | **Prevalence** | **Rate** | **PAR** | **Relative risk** | **95%CI** |
|  | **(n)** | **(n)** | **(%)** | **per 1000** | **(%)** | **(n)** | **(n)** |
| Australia | 15,880 | 150 | 1.3 | 9.4 | 6.2 | 21.9 | (18.2, 26.4) |
| Brazil | 369,804 | 17,641 | 1.6 | 47.7 | 10.8 | 20.3 | (20.0, 20.7) |
| Canada | 55,647 | 558 | 1.3 | 10.0 | 6.4 | 31.7 | (28.7, 35.1) |
| Czech Republic | 1,587 | 13 | 1.4 | 8.2 | 7.2 | 18.2 | (9.8, 33.8) |
| Denmark | 12,580 | 162 | 1.1 | 12.9 | 6.6 | 16.5 | (13.9, 19.6) |
| England and Wales | 43,704 | 576 | 1.4 | 13.2 | 8.1 | 18.6 | (17.0, 20.4) |
| Estonia | 724 | 9 | 0.9 | 12.4 | 9.4 | 29.2 | (13.8, 61.8) |
| Lebanon | 478 | 7 | 1.8 | 14.6 | 6.0 | 8.3 | (3.7, 18.4) |
| Mexico | 64,898 | 1,395 | 1.2 | 21.5 | 3.1 | 4.1 | (3.9, 4.4) |
| Netherlands | 22,611 | 370 | 1.2 | 16.4 | 6.3 | 19.8 | (17.7, 22.2) |
| Qatar | 1,765 | 33 | 1.9 | 18.7 | 11.8 | 27.1 | (17.6, 41.5) |
| Scotland | 16,500 | 214 | 1.5 | 13.0 | 8.1 | 16.5 | (14.2, 19.3) |
| Sweden | 18,145 | 271 | 0.9 | 14.9 | 9.6 | 24.8 | (21.7, 28.4) |
| Uruguay | 7,905 | 66 | 1.6 | 8.3 | 9.9 | 22.8 | (17.1, 30.4) |
| USA | 1,275,412 | 21,118 | 1.6 | 16.6 | 7.2 | 21.4 | (21.0, 21.7) |

| Country | **2,000g to 2,500g** | | | | | | |
| --- | --- | --- | --- | --- | --- | --- | --- |
|  | **Livebirths** | **Deaths** | **Prevalence** | **Rate** | **PAR** | **Relative risk** | **95%CI** |
|  | **(n)** | **(n)** | **(%)** | **per 1000** | **(%)** | **(n)** | **(n)** |
| Australia | 53,099 | 154 | 4.4 | 2.9 | 5.7 | 6.8 | (5.6, 8.1) |
| Brazil | 1,299,612 | 16,610 | 5.5 | 12.8 | 9.1 | 5.6 | (5.5, 5.7) |
| Canada | 183,318 | 490 | 4.1 | 2.7 | 5.2 | 8.5 | (7.7, 9.5) |
| Czech Republic | 4,893 | 12 | 4.4 | 2.5 | 5.8 | 5.5 | (2.9, 10.4) |
| Denmark | 35,438 | 166 | 3.2 | 4.7 | 6.1 | 6.1 | (5.1, 7.2) |
| England and Wales | 154,349 | 639 | 4.8 | 4.1 | 8.0 | 5.9 | (5.4, 6.5) |
| Estonia | 2,089 | 11 | 2.5 | 5.3 | 11.0 | 12.5 | (6.2, 25.1) |
| Lebanon | 1,532 | 6 | 5.8 | 3.9 | 3.3 | 2.2 | (0.9, 5.3) |
| Mexico | 266,439 | 2,290 | 4.8 | 8.6 | 2.7 | 1.7 | (1.6, 1.7) |
| Netherlands | 70,134 | 370 | 3.8 | 5.3 | 5.6 | 6.5 | (5.7, 7.2) |
| Qatar | 5,712 | 33 | 6.1 | 5.8 | 11.0 | 8.5 | (5.5, 13.0) |
| Scotland | 49,971 | 228 | 4.4 | 4.6 | 7.7 | 5.9 | (5.1, 6.8) |
| Sweden | 54,417 | 312 | 2.6 | 5.7 | 10.5 | 9.6 | (8.5, 10.9) |
| Uruguay | 25,043 | 45 | 5.0 | 1.8 | 5.7 | 4.9 | (3.5, 6.9) |
| USA | 4,075,989 | 21,953 | 5.1 | 5.4 | 6.8 | 7.0 | (6.9, 7.1) |

| **Country** | **4,000 to 4,500g** | | | | | | |
| --- | --- | --- | --- | --- | --- | --- | --- |
|  | **Livebirths** | **Deaths** | **Prevalence** | **Rate** | **PAR** | **Relative risk** | **95%CI** |
|  | **(n)** | **(n)** | **(%)** | **per 1000** | **(%)** | **(n)** | **(n)** |
| Australia | 102,107 | 19 | 8.4 | 0.2 | -1.1 | 0.4 | (0.3, 0.7) |
| Brazil | 1,044,426 | 2,205 | 4.5 | 2.1 | -0.1 | 0.9 | (0.9, 1.0) |
| Canada | 414,097 | 107 | 9.3 | 0.3 | -0.3 | 0.8 | (0.7, 1.0) |
| Czech Republic | 8,253 | 1 | 7.4 | 0.1 | -1.6 | 0.3 | (0.0, 2.0) |
| Denmark | 159,341 | 75 | 14.5 | 0.5 | -2.1 | 0.6 | (0.5, 0.8) |
| England and Wales | 295,023 | 136 | 9.2 | 0.5 | -1.1 | 0.7 | (0.6, 0.8) |
| Estonia | 12,516 | 2 | 15.2 | 0.2 | -3.5 | 0.4 | (0.1, 1.6)) |
| Lebanon | 1,026 | 1 | 3.9 | 1.0 | -0.8 | 0.6 | (0.1, 4.1) |
| Mexico | 148,582 | 748 | 2.7 | 5.0 | 0.0 | 1.0 | (0.9, 1.1) |
| Netherlands | 213,935 | 108 | 11.5 | 0.5 | -1.2 | 0.6 | (0.5, 0.8) |
| Qatar | 4,197 | 3 | 4.4 | 0.7 | 0.1 | 1.1 | (0.3, 3.4) |
| Scotland | 124,876 | 54 | 11.1 | 0.4 | -1.8 | 0.6 | (0.4, 0.7) |
| Sweden | 314,857 | 129 | 15.0 | 0.4 | -2.2 | 0.7 | (0.6, 0.8) |
| Uruguay | 34,036 | 9 | 6.8 | 0.3 | -0.5 | 0.7 | (0.4, 1.4) |
| USA | 5,654,779 | 2,705 | 7.0 | 0.5 | -0.6 | 0.6 | (0.6, 0.7) |

| **Country** | **4,500g to 5,000g** | | | | | | |
| --- | --- | --- | --- | --- | --- | --- | --- |
|  | **Livebirths** | **Deaths** | **Prevalence** | **Rate** | **PAR** | **Relative risk** | **95%CI** |
|  | **(n)** | **(n)** | **(%)** | **per 1000** | **(%)** | **(n)** | **(n)** |
| Australia | 13,218 | 12 | 1.1 | 0.9 | 0.3 | 2.1 | (1.2, 3.8) |
| Brazil | 130,339 | 544 | 0.6 | 4.2 | 0.2 | 1.9 | (1.7, 2.0) |
| Canada | 67,473 | 54 | 1.5 | 0.8 | 0.4 | 2.6 | (1.9, 3.4) |
| Czech Republic | 864 | 1 | 0.8 | 1.2 | 0.4 | 2.6 | (0.4, 18.8) |
| Denmark | 31,315 | 23 | 2.8 | 0.7 | 0.0 | 1.0 | (1.4, 0.6) |
| England and Wales | 41,966 | 29 | 1.3 | 0.7 | 0.0 | 1.0 | (0.7, 1.4) |
| Estonia | 2,019 | 1 | 2.5 | 0.5 | 0.2 | 1.2 | (0.2, 8.7) |
| Lebanon | 117 | 1 | 0.4 | 8.5 | 0.8 | 4.9 | (0.7, 35.0) |
| Mexico | 2,347 | 13 | 0.0 | 5.5 | 0.0 | 1.1 | (0.6, 1.9) |
| Netherlands | 32,974 | 14 | 1.8 | 0.4 | -0.2 | 0.5 | (0.3, 0.9) |
| Qatar | 397 | 0 | 0.4 | 0.0 | -0.1 | 0.0 | (0, 0) |
| Scotland | 20,666 | 15 | 1.8 | 0.7 | 0.0 | 0.9 | (0.6, 1.6) |
| Sweden | 66,053 | 41 | 3.1 | 0.6 | 0.1 | 1.0 | (0.8, 1.4) |
| Uruguay | 4,132 | 3 | 0.8 | 0.7 | 0.2 | 2.0 | (0.6, 6.3) |
| USA | 819,941 | 755 | 1.0 | 0.9 | 0.0 | 1.2 | (1.1, 1.3) |

| **Country** | **>5,000g** | | | | | | |
| --- | --- | --- | --- | --- | --- | --- | --- |
|  | **Livebirths** | **Deaths** | **Prevalence** | **Rate** | **PAR** | **Relative risk** | **95%CI** |
|  | **(n)** | **(n)** | **(%)** | **per 1000** | **(%)** | **(n)** | **(n)** |
| Australia | 1,085 | 8 | 0.1 | 7.4 | 0.3 | 17.1 | (8.5, 34.4) |
| Brazil | 17,477 | 276 | 0.1 | 15.8 | 0.2 | 6.9 | (6.2, 7.8) |
| Canada | 8,781 | 52 | 0.2 | 5.9 | 0.6 | 18.8 | (14.3, 24.8) |
| Czech Republic | 56 | 0 | 0.0 | 0.0 | 0.0 | 0.0 | (0,0) |
| Denmark | 4,023 | 5 | 0.4 | 1.2 | 0.1 | 1.6 | (0.7, 3.9)) |
| England and Wales | 3,725 | 6 | 0.1 | 1.6 | 0.1 | 2.3 | (1.0, 5.1) |
| Estonia | 186 | 0 | 0.2 | 0.0 | -0.1 | 0.0 | (0,0) |
| Lebanon | 13 | 0 | 0.0 | 0.0 | 0.0 | 0.0 | (0,0) |
| Mexico | 223 | 0 | 0.0 | 0.0 | 0.0 | 0.0 | (0,0) |
| Netherlands | 2,929 | 9 | 0.2 | 3.1 | 0.1 | 3.8 | (2.0, 7.2) |
| Qatar | 30 | 0 | 0.0 | 0.0 | 0.0 | 0.0 | (0,0) |
| Scotland | 2,251 | 2 | 0.2 | 0.9 | 0.0 | 1.1 | (0.3, 4.6) |
| Sweden | 8,824 | 8 | 0.4 | 0.9 | 0.1 | 1.5 | (0.8, 3.1) |
| Uruguay | 397 | 0 | 0.1 | 0.0 | 0.0 | 0.0 | (0,0) |
| USA | 92,370 | 307 | 0.1 | 3.3 | 0.1 | 4.3 | (3.9, 4.9) |

# **Table S9. Neonatal mortality NMR, population attributable risk, and crude relative risk of neonatal mortality for gestational age fine strata (reference 37 to 42 completed weeks), by country**

Prevalence = the number of livebirths reported in each group of interest / total number of livebirths

Neonatal Mortality NMR (NMR): the number of persons who experienced the event (neonatal death) divided by the total number of persons exposed to the risk of that event per 1000

Population attributable risk (PAR): was calculated for each level of exposure with the following formula where *pr* is the prevalence and *RR* is the relative risk ^8^

$PAR for type of interest= \frac{pr\left( type of interest \right)\left( RR\left( type of interest-1 \right) \right)}{\sum_{all types} pr\left( type \right)x RR(type)}$

Relative risk: the absolute risk within a specific category divided by the absolute risk in the reference group, expressed as a ratio

| **Country** | **Total (all groups)** | | | **37 to 42 weeks (reference group)** | | | |
| --- | --- | --- | --- | --- | --- | --- | --- |
|  | **Livebirths** | **Deaths** | **Rate** | **Livebirths** | **Deaths** | **Prevalence** | **Rate** |
|  | **(n)** | **(n)** | **per 1000** | **(n)** | **(n)** | **(%)** | **per 1000** |
| Australia | 1,216,690 | 2,166 | 1.8 | 1,118,699 | 435 | 91.95 | 0.4 |
| Brazil | 21,355,008 | 149,234 | 7.0 | 18,665,492 | 45,531 | 87.41 | 2.4 |
| Canada | 4,386,246 | 7,794 | 1.8 | 4,030,983 | 1,281 | 91.90 | 0.3 |
| Czech Republic | 109,492 | 168 | 1.5 | 101,779 | 47 | 92.96 | 0.5 |
| Denmark | 1,100,854 | 2,598 | 2.4 | 1,026,140 | 697 | 93.21 | 0.7 |
| England and Wales | 3,212,492 | 7,169 | 2.2 | 2,966,254 | 2,032 | 92.33 | 0.7 |
| Estonia | 82,427 | 95 | 1.2 | 76,091 | 33 | 92.31 | 0.4 |
| Lebanon | 25,966 | 123 | 4.7 | 23,345 | 39 | 89.91 | 1.7 |
| Mexico | 5,560,916 | 34,036 | 6.1 | 5,177,919 | 26,278 | 93.11 | 5.1 |
| Netherlands | 1,849,432 | 5,556 | 3.0 | 1,719,661 | 1,269 | 92.98 | 0.7 |
| Qatar | 94,337 | 297 | 3.1 | 84,793 | 71 | 89.88 | 0.8 |
| Scotland | 1,126,199 | 2,643 | 2.3 | 1,039,449 | 764 | 92.30 | 0.7 |
| Sweden | 2,102,671 | 2,755 | 1.3 | 1,981,034 | 1,079 | 94.22 | 0.5 |
| Uruguay | 493,789 | 670 | 1.4 | 448,592 | 156 | 90.85 | 0.3 |
| USA | 80,234,505 | 253,275 | 3.2 | 72,064,969 | 55,978 | 89.82 | 0.8 |

| **Country** | **<28 weeks (extremely preterm)** | | | | | | |
| --- | --- | --- | --- | --- | --- | --- | --- |
|  | **Livebirths** | **Deaths** | **Prevalence** | **Rate** | **PAR** | **Relative risk** | **95%CI** |
|  | **(n)** | **(n)** | **(%)** | **per 1000** | **(%)** | **(n)** |  |
| Australia | 4,612 | 1,243 | 0.4 | 269.5 | 51.6 | 546.2 | (491.2, 607.3) |
| Brazil | 107,879 | 46,232 | 0.5 | 428.6 | 24.5 | 123.3 | (121.8, 124.8) |
| Canada | 17,699 | 4,835 | 0.4 | 273.2 | 56.3 | 675.4 | (635.9, 717.3) |
| Czech Republic | 319 | 75 | 0.3 | 235.1 | 39.6 | 412.4 | (290.3, 585.8) |
| Denmark | 3,522 | 1,266 | 0.3 | 359.5 | 41.3 | 389.5 | (425.3, 356.7)) |
| England and Wales | 15,930 | 3,248 | 0.5 | 203.9 | 40.8 | 247.4 | (234.5, 261.0) |
| Estonia | 299 | 41 | 0.4 | 137.1 | 40.4 | 278.2 | (178.1, 434.4) |
| Lebanon | 70 | 38 | 0.3 | 542.9 | 22.9 | 211.0 | (140.7, 316.2) |
| Mexico | 11,674 | 2,056 | 0.2 | 176.1 | 5.1 | 29.7 | (28.4, 30.9) |
| Netherlands | 7,532 | 3,012 | 0.4 | 399.9 | 46.0 | 387.4 | (363.8, 412.4) |
| Qatar | 501 | 137 | 0.5 | 273.5 | 40.2 | 256.7 | (194.8, 338.2) |
| Scotland | 3,977 | 1,137 | 0.4 | 285.9 | 37.1 | 302.7 | (277.4, 330.4) |
| Sweden | 5,249 | 718 | 0.2 | 136.8 | 23.7 | 221.0 | (201.8, 242.1) |
| Uruguay | 1,926 | 291 | 0.4 | 151.1 | 40.1 | 377.6 | (312.2, 456.6) |
| USA | 479,983 | 132,963 | 0.6 | 277.0 | 46.5 | 279.5 | (276.8, 282.1) |

| **Country** | **28 to 31 weeks (very preterm)** | | | | | | |
| --- | --- | --- | --- | --- | --- | --- | --- |
|  | **Livebirths** | **Deaths** | **Prevalence** | **Rate** | **PAR** | **Relative risk** | **95%CI** |
|  | **(n)** | **(n)** | **(%)** | **per 1000** | **(%)** | **(n)** | **(n)** |
| Australia | 8,190 | 179 | 0.7 | 21.9 | 9.1 | 55.0 | (46.3, 65.4) |
| Brazil | 227,340 | 26,450 | 1.1 | 116.3 | 17.6 | 42.8 | (42.2, 43.5) |
| Canada | 31,288 | 691 | 0.7 | 22.1 | 9.9 | 68.0 | (62.0, 74.6) |
| Czech Republic | 722 | 17 | 0.7 | 23.5 | 10.6 | 49.8 | (28.8, 86.4) |
| Denmark | 7,933 | 279 | 0.7 | 35.2 | 11.8 | 50.1 | (43.6, 57.4) |
| England and Wales | 23,518 | 783 | 0.7 | 33.3 | 11.3 | 47.1 | (43.4, 51.1) |
| Estonia | 465 | 6 | 0.6 | 12.9 | 6.4 | 29.4 | (12.4, 69.8) |
| Lebanon | 183 | 22 | 0.7 | 120.2 | 18.1 | 64.3 | (38.9, 106.5) |
| Mexico | 26,385 | 1,743 | 0.5 | 66.1 | 4.5 | 12.3 | (11.7, 12.9) |
| Netherlands | 13,017 | 541 | 0.7 | 41.6 | 10.9 | 54.1 | (49.0, 59.8) |
| Qatar | 950 | 21 | 1.0 | 22.1 | 7.4 | 25.9 | (15.9, 41.9) |
| Scotland | 9,356 | 336 | 0.8 | 35.9 | 13.4 | 47.2 | (41.6, 53.6) |
| Sweden | 12,263 | 377 | 0.6 | 30.7 | 13.5 | 54.8 | (48.8, 61.5) |
| Uruguay | 4,397 | 105 | 0.9 | 23.9 | 16.1 | 67.1 | (52.5, 85.8) |
| USA | 763,621 | 24,779 | 1.0 | 32.4 | 10.5 | 40.5 | (39.9, 41.1) |

| **Country** | **32 to 33 weeks (moderate preterm)** | | | | | | |
| --- | --- | --- | --- | --- | --- | --- | --- |
|  | **Livebirths** | **Deaths** | **Prevalence** | **Rate** | **PAR** | **Relative risk** | **95%CI** |
|  | **(n)** | **(n)** | **(%)** | **per 1000** | **(%)** | **(n)** | **(n)** |
| Australia | 11,434 | 94 | 0.9 | 8.2 | 4.7 | 21.0 | (16.8, 26.2) |
| Brazil | 341,967 | 11,587 | 1.6 | 33.9 | 7.9 | 13.5 | (13.2, 13.7) |
| Canada | 42,074 | 350 | 1.0 | 8.3 | 5.0 | 26.0 | (23.1, 29.2) |
| Czech Republic | 1,018 | 16 | 0.9 | 15.7 | 10.0 | 33.5 | (19.1, 58.9) |
| Denmark | 10,369 | 114 | 0.9 | 11.0 | 4.7 | 16.0 | (13.2, 19.5) |
| England and Wales | 27,287 | 372 | 0.8 | 13.6 | 5.3 | 19.6 | (17.6, 21.9) |
| Estonia | 567 | 8 | 0.7 | 14.1 | 8.6 | 32.1 | (14.9, 69.2) |
| Lebanon | 304 | 9 | 1.2 | 29.6 | 7.7 | 17.2 | (8.4, 35.3) |
| Mexico | 41,644 | 1,035 | 0.7 | 24.9 | 2.4 | 4.8 | (4.5, 5.1) |
| Netherlands | 16,496 | 270 | 0.9 | 16.4 | 5.4 | 21.8 | (19.1, 24.9) |
| Qatar | 1,153 | 21 | 1.2 | 18.2 | 7.4 | 21.4 | (13.2, 34.7) |
| Scotland | 11,477 | 145 | 1.0 | 12.6 | 5.7 | 17.0 | (14.2, 20.3) |
| Sweden | 15,862 | 200 | 0.8 | 12.6 | 7.1 | 22.9 | (19.7, 26.6) |
| Uruguay | 5,510 | 43 | 1.1 | 7.8 | 6.5 | 22.3 | (15.9, 31.2) |
| USA | 967,381 | 12,928 | 1.2 | 13.4 | 5.4 | 17.0 | (16.7, 17.3) |

| **Country** | **34 to 36 weeks (late preterm)** | | | | | | |
| --- | --- | --- | --- | --- | --- | --- | --- |
|  | **Livebirths** | **Deaths** | **Prevalence** | **Rate** | **PAR** | **Relative risk** | **95%CI** |
|  | **(n)** | **(n)** | **(%)** | **per 1000** | **(%)** | **(n)** | **(n)** |
| Australia | 73,573 | 211 | 6.0 | 2.9 | 9.6 | 7.4 | (6.2, 8.7) |
| Brazil | 1,792,906 | 18,620 | 8.4 | 10.4 | 10.7 | 4.2 | (4.2, 4.3) |
| Canada | 263,820 | 637 | 6.0 | 2.4 | 8.2 | 7.6 | (6.9, 8.3) |
| Czech Republic | 5,468 | 13 | 5.0 | 2.4 | 6.8 | 5.1 | (2.8, 9.5) |
| Denmark | 51,751 | 239 | 4.7 | 4.6 | 9.0 | 6.8 | (5.8, 7.8) |
| England and Wales | 176,704 | 733 | 5.5 | 4.1 | 9.3 | 6.0 | (5.5, 6.6) |
| Estonia | 3,436 | 6 | 4.2 | 1.7 | 5.1 | 4.0 | (1.7, 9.6) |
| Lebanon | 2,053 | 15 | 7.9 | 7.3 | 10.7 | 4.3 | (2.4, 7.9) |
| Mexico | 302,785 | 2,922 | 5.4 | 9.7 | 4.1 | 1.9 | (1.8, 2.0) |
| Netherlands | 92,464 | 464 | 5.0 | 5.0 | 8.4 | 6.8 | (6.1, 7.5) |
| Qatar | 6,854 | 47 | 7.3 | 6.9 | 15.4 | 8.1 | (5.6, 11.8) |
| Scotland | 61,288 | 260 | 5.4 | 4.2 | 9.0 | 5.8 | (5.0, 6.6) |
| Sweden | 88,263 | 381 | 4.2 | 4.3 | 12.5 | 7.9 | (7.0, 8.9) |
| Uruguay | 33,341 | 75 | 6.8 | 2.2 | 10.1 | 6.5 | (4.9, 8.5) |
| USA | 5,831,509 | 26,407 | 7.3 | 4.5 | 9.8 | 5.8 | (5.7, 5.9) |

| **Country** | **>42 weeks** | | | | | | |
| --- | --- | --- | --- | --- | --- | --- | --- |
|  | **Livebirths** | **Deaths** | **Prevalence** | **Rate** | **PAR** | **Relative risk** | **95%CI** |
|  | **(n)** | **(n)** | **(%)** | **per 1000** | **(%)** | **(n)** | **(n)** |
| Australia | 182 | Not provided | | | | | |
| Brazil | 219,424 | 814 | 0.0 | 3.7 | 0.0 | 1.5 | (1.4, 1.6) |
| Canada | 382 | 0 | 0.0 | 0.0 | 0.0 | 0.0 | (0, 0) |
| Czech Republic | 186 | 0 | 0.0 | 0.0 | 0.0 | 0.0 | (0, 0) |
| Denmark | 1,139 | 0 | 0.0 | 0.0 | 0.0 | 0.0 | (0, 0) |
| England and Wales | 2,799 | 1 | 0.0 | 0.4 | 0.0 | 0.5 | (0.1, 3.7) |
| Estonia | 1,569 | 1 | 0.0 | 0.6 | 0.0 | 1.5 | (0.2, 10.7) |
| Lebanon | 11 | 0 | 0.0 | 0.0 | 0.0 | 0.0 | (0, 0) |
| Mexico | 509 | 2 | 0.0 | 3.9 | 0.0 | 0.8 | (0.2, 3.1) |
| Netherlands | 262 | 0 | 0.0 | 0.0 | 0.0 | 0.0 | (0, 0) |
| Qatar | 86 | 0 | 0.0 | 0.0 | 0.0 | 0.0 | (0, 0) |
| Scotland | 652 | 1 | 0.0 | 1.5 | 0.0 | 2.1 | (0.3, 14.8) |
| Sweden | 0 | 0 | 0.0 | 0.0 | 0.0 | 0.0 | (0, 0) |
| Uruguay | 23 | 0 | 0.0 | 0.0 | 0.0 | 0.0 | (0, 0) |
| USA | 127,042 | 220 | 0.0 | 1.7 | 0.0 | 2.2 | 2.0, 2.5) |

# **Table S10. Neonatal mortality NMR, population attributable risk, and crude relative risk of neonatal mortality for 6 newborn types (reference T+AGA), by country**

Prevalence = the number of livebirths reported in each group of interest / total number of livebirths

Neonatal Mortality NMR (NMR): the number of persons who experienced the event (neonatal death) divided by the total number of persons exposed to the risk of that event per 1000

Population attributable risk (PAR): was calculated for each level of exposure with the following formula where *pr* is the prevalence and *RR* is the relative risk ^8^

$PAR for type of interest= \frac{pr\left( type of interest \right)\left( RR\left( type of interest-1 \right) \right)}{\sum_{all types} pr\left( type \right)x RR(type)}$

Relative risk: the absolute risk within a specific category divided by the absolute risk in the reference group, expressed as a ratio

| **Country** | **Total** | | **T+AGA** | | | |
| --- | --- | --- | --- | --- | --- | --- |
|  | **Livebirths** | **Deaths** | **Livebirths** | **Deaths** | **Prevalence** | **Rate** |
|  | **(n)** | **(n)** | **(n)** | **(n)** | **(%)** | **per 1000** |
| Australia | 1,216,172 | 2,096 | 836,833 | 288 | 68.8 | 0.3 |
| Brazil | 21,345,439 | 148,418 | 14,678,798 | 27,030 | 68.8 | 1.8 |
| Canada | 4,388,387 | 10,000 | 2,970,502 | 748 | 67.7 | 0.3 |
| Czech Republic | 109,492 | 168 | 80,651 | 32 | 73.7 | 0.4 |
| Denmark | 1,100,854 | 2,598 | 709,716 | 446 | 64.5 | 0.6 |
| England and Wales | 3,212,492 | 7,169 | 2,251,910 | 1,342 | 70.1 | 0.6 |
| Estonia | 82,298 | 95 | 52,294 | 20 | 63.5 | 0.4 |
| Lebanon | 25,515 | 114 | 18,280 | 25 | 71.6 | 1.4 |
| Mexico | 5,560,916 | 34,036 | 4,342,347 | 21,801 | 78.1 | 5.0 |
| Netherlands | 1,846,558 | 5,483 | 1,235,592 | 812 | 66.9 | 0.7 |
| Qatar | 94,337 | 297 | 67,589 | 42 | 71.6 | 0.6 |
| Scotland | 1,124,817 | 2,599 | 757,774 | 497 | 67.4 | 0.7 |
| Sweden | 2,102,671 | 2,755 | 1,412,703 | 668 | 67.2 | 0.5 |
| Uruguay | 493,040 | 664 | 341,543 | 109 | 69.3 | 0.3 |
| USA | 80,225,526 | 252,745 | 54,381,179 | 33,640 | 67.8 | 0.6 |

| **Country** | **PT+SGA** | | | | | | | |
| --- | --- | --- | --- | --- | --- | --- | --- | --- |
|  | **Livebirths** | **Deaths** | **Prevalence** | **Rate** | **PAR** | **RR** | **95%CI** | |
|  | **(n)** | **(n)** | **(%)** | **per 1000** | **(%)** | **(n)** | **(n)** | |
| Australia | 7,747 | 186 | 0.6 | 24.0 | 8.6 | 68.2 | 56.8 | 81.8 |
| Brazil | 184,780 | 23,092 | 0.9 | 125.0 | 14.2 | 60.4 | 59.4 | 61.5 |
| Canada | 26,860 | 766 | 0.6 | 28.5 | 7.5 | 110.1 | 99.7 | 121.7 |
| Czech Republic | 730 | 20 | 0.7 | 27.4 | 11.6 | 67.2 | 38.6 | 117.0 |
| Denmark | 5,422 | 285 | 0.5 | 52.6 | 10.5 | 79.5 | 68.7 | 92.0 |
| England and Wales | 27,402 | 662 | 0.9 | 24.2 | 8.9 | 39.6 | 36.1 | 43.4 |
| Estonia | 286 | 2 | 0.3 | 7.0 | 1.2 | 6.0 | 1.5 | 24.3 |
| Lebanon | 271 | 14 | 1.1 | 51.7 | 11.6 | 36.0 | 18.9 | 68.5 |
| Mexico | 37,018 | 790 | 0.7 | 21.3 | 1.7 | 4.2 | 3.9 | 4.5 |
| Netherlands | 10,899 | 528 | 0.6 | 48.4 | 9.3 | 70.4 | 63.2 | 78.4 |
| Qatar | 863 | 43 | 0.9 | 49.8 | 13.9 | 76.4 | 50.2 | 116.3 |
| Scotland | 7,786 | 273 | 0.7 | 35.1 | 10.1 | 51.7 | 44.7 | 59.8 |
| Sweden | 10,807 | 346 | 0.5 | 32.0 | 12.1 | 65.6 | 57.7 | 74.6 |
| Uruguay | 3,566 | 83 | 0.7 | 23.3 | 12.2 | 71.3 | 53.7 | 94.7 |
| USA | 584,823 | 34,019 | 0.7 | 58.2 | 12.9 | 88.9 | 87.6 | 90.3 |

| **Country** | **PT+AGA** | | | | | | | |
| --- | --- | --- | --- | --- | --- | --- | --- | --- |
|  | **Livebirths** | **Deaths** | **Prevalence** | **Rate** | **PAR** | **RR** | **95%CI** | |
|  | **(n)** | **(n)** | **(%)** | **per 1000** | **(%)** | **(n)** | **(n)** | |
| Australia | 77,760 | 1,304 | 6.4 | 16.8 | 60.2 | 47.9 | 42.1 | 54.4 |
| Brazil | 1,491,029 | 66,036 | 7.0 | 44.3 | 42.4 | 23.1 | 22.8 | 23.4 |
| Canada | 280,730 | 7,258 | 6.4 | 25.9 | 71.6 | 100.1 | 92.9 | 107.9 |
| Czech Republic | 6,177 | 93 | 5.6 | 15.1 | 53.7 | 37.4 | 25.0 | 55.8 |
| Denmark | 57,053 | 1,382 | 5.2 | 24.2 | 51.6 | 37.7 | 33.9 | 41.9 |
| England and Wales | 191,142 | 3,987 | 5.9 | 20.9 | 53.7 | 34.3 | 32.3 | 36.5 |
| Estonia | 3,774 | 46 | 4.6 | 12.2 | 30.6 | 10.4 | 7.4 | 14.8 |
| Lebanon | 1,825 | 56 | 7.2 | 30.7 | 46.5 | 21.8 | 13.6 | 34.8 |
| Mexico | 319,589 | 6,586 | 5.7 | 20.6 | 14.4 | 4.0 | 3.9 | 4.2 |
| Netherlands | 102,958 | 3,272 | 5.6 | 31.8 | 58.1 | 46.9 | 43.4 | 50.6 |
| Qatar | 7,290 | 166 | 7.7 | 22.8 | 54.2 | 35.9 | 25.6 | 50.3 |
| Scotland | 67,902 | 1,363 | 6.0 | 20.1 | 50.5 | 30.0 | 27.1 | 33.3 |
| Sweden | 97,940 | 1,155 | 4.7 | 11.8 | 40.2 | 24.7 | 22.4 | 27.1 |
| Uruguay | 36,379 | 381 | 7.4 | 10.5 | 55.6 | 32.5 | 26.3 | 40.2 |
| USA | 6,165,319 | 140,547 | 7.7 | 22.8 | 54.0 | 36.1 | 35.6 | 36.5 |

| **Country** | **PT+LGA** | | | | | | | |
| --- | --- | --- | --- | --- | --- | --- | --- | --- |
|  | **Livebirths** | **Deaths** | **Prevalence** | **Rate** | **PAR (%)** | **RR** | **95%CI** | |
|  | **(n)** | **(n)** | **(%)** | **per 1000** | **(%)** | **(n)** | **(n)** | |
| Australia | 12,215 | 201 | 1.0 | 16.5 | 9.3 | 47.1 | 39.3 | 56.3 |
| Brazil | 791,532 | 13,194 | 3.7 | 16.7 | 8.1 | 8.9 | 8.7 | 9.1 |
| Canada | 49,522 | 695 | 1.1 | 14.0 | 6.9 | 55.0 | 49.6 | 60.9 |
| Czech Republic | 620 | 8 | 0.6 | 12.9 | 4.6 | 32.1 | 14.9 | 69.4 |
| Denmark | 11,100 | 231 | 1.0 | 20.8 | 8.6 | 32.5 | 27.7 | 38.0 |
| England and Wales | 24,895 | 487 | 0.8 | 19.6 | 6.6 | 32.2 | 29.1 | 35.7 |
| Estonia | 691 | 13 | 0.8 | 18.8 | 8.9 | 16.0 | 9.0 | 28.5 |
| Lebanon | 449 | 10 | 1.8 | 22.3 | 8.2 | 16.0 | 7.7 | 33.0 |
| Mexico | 25,881 | 380 | 0.5 | 14.7 | 0.7 | 2.9 | 2.6 | 3.2 |
| Netherlands | 15,425 | 425 | 0.8 | 27.6 | 7.5 | 40.8 | 36.3 | 45.9 |
| Qatar | 1,305 | 17 | 1.4 | 13.0 | 5.5 | 20.7 | 11.8 | 36.3 |
| Scotland | 10,086 | 210 | 0.9 | 20.8 | 7.8 | 31.1 | 26.5 | 36.5 |
| Sweden | 12,890 | 175 | 0.6 | 13.6 | 6.1 | 28.3 | 24.0 | 33.4 |
| Uruguay | 5,109 | 46 | 1.0 | 9.0 | 6.7 | 28.0 | 19.8 | 39.4 |
| USA | 1,289,830 | 22,074 | 1.6 | 17.1 | 8.5 | 27.2 | 26.8 | 27.7 |

| **Country** | **T+SGA** | | | | | | | |
| --- | --- | --- | --- | --- | --- | --- | --- | --- |
|  | **Livebirths** | **Deaths** | **Prevalence** | **Rate** | **PAR** | **RR** | **95%CI** | |
|  | **(n)** | **(n)** | **(%)** | **per 1000** | **(%)** | **(n)** | **(n)** | |
| Australia | 34,081 | 53 | 2.8 | 1.6 | 2.8 | 6.0 | 4.6 | 7.7 |
| Brazil | 1,524,445 | 14,494 | 7.1 | 9.5 | 8.1 | 5.1 | 5.0 | 5.2 |
| Canada | 142,368 | 341 | 3.2 | 2.4 | 3.1 | 9.5 | 8.4 | 10.8 |
| Czech Republic | 5,583 | 12 | 5.1 | 2.1 | 5.9 | 5.4 | 2.8 | 10.5 |
| Denmark | 32,092 | 113 | 2.9 | 3.5 | 3.6 | 5.6 | 4.5 | 6.9 |
| England and Wales | 133,988 | 426 | 4.2 | 3.2 | 4.9 | 5.3 | 4.8 | 5.9 |
| Estonia | 1,524 | 8 | 1.9 | 5.2 | 4.6 | 4.5 | 2.2 | 9.3 |
| Lebanon | 1,585 | 6 | 6.2 | 3.8 | 3.4 | 2.8 | 1.1 | 6.7 |
| Mexico | 371,565 | 2,194 | 6.7 | 5.9 | 1.0 | 1.2 | 1.1 | 1.2 |
| Netherlands | 56,877 | 237 | 3.1 | 4.2 | 3.7 | 6.3 | 5.5 | 7.3 |
| Qatar | 5,349 | 23 | 5.7 | 4.3 | 6.7 | 6.9 | 4.1 | 11.5 |
| Scotland | 50,411 | 144 | 4.5 | 2.9 | 4.3 | 4.3 | 3.6 | 5.2 |
| Sweden | 69,761 | 208 | 3.3 | 3.0 | 6.4 | 6.3 | 5.4 | 7.3 |
| Uruguay | 18,853 | 24 | 3.8 | 1.3 | 2.7 | 4.0 | 2.6 | 6.2 |
| USA | 3,258,384 | 15,695 | 4.1 | 4.8 | 5.5 | 7.8 | 7.6 | 7.9 |

| **Country** | **T+LGA** | | | | | | | |
| --- | --- | --- | --- | --- | --- | --- | --- | --- |
|  | **Livebirths** | **Deaths** | **Prevalence** | **Rate** | **PAR** | **RR** | **95%CI** | |
|  | **(n)** | **(n)** | **(%)** | **per 1000** | **(%)** | **(n)** | **(n)** | |
| Australia | 247,536 | 64 | 20.4 | 0.3 | -1.0 | 0.8 | 0.6 | 1.0 |
| Brazil | 2,674,855 | 4,572 | 12.5 | 1.7 | -0.2 | 0.9 | 0.9 | 1.0 |
| Canada | 918,405 | 192 | 20.9 | 0.2 | -0.4 | 0.8 | 0.7 | 1.0 |
| Czech Republic | 15,731 | 3 | 14.4 | 0.2 | -2.0 | 0.5 | 0.1 | 1.6 |
| Denmark | 285,471 | 141 | 25.9 | 0.5 | -1.5 | 0.8 | 0.7 | 0.9 |
| England and Wales | 583,155 | 265 | 18.2 | 0.5 | -1.2 | 0.8 | 0.7 | 0.9 |
| Estonia | 23,729 | 6 | 28.8 | 0.3 | -15.9 | 0.2 | 0.1 | 0.5 |
| Lebanon | 3,105 | 3 | 12.2 | 1.0 | -1.1 | 0.7 | 0.2 | 2.3 |
| Mexico | 464,516 | 2,285 | 8.4 | 4.9 | -0.1 | 1.0 | 0.9 | 1.0 |
| Netherlands | 424,807 | 209 | 23.0 | 0.5 | -1.3 | 0.7 | 0.6 | 0.9 |
| Qatar | 11,941 | 6 | 12.7 | 0.5 | -0.5 | 0.8 | 0.3 | 1.9 |
| Scotland | 230,858 | 112 | 20.5 | 0.5 | -1.5 | 0.7 | 0.6 | 0.9 |
| Sweden | 498,570 | 203 | 23.7 | 0.4 | -1.2 | 0.9 | 0.7 | 1.0 |
| Uruguay | 87,590 | 21 | 17.8 | 0.2 | -1.1 | 0.8 | 0.5 | 1.2 |
| USA | 14,545,991 | 6,770 | 18.1 | 0.5 | -0.9 | 0.8 | 0.7 | 0.8 |

# **Table S11. Neonatal mortality NMR, population attributable risk, and crude relative risk of neonatal mortality for 10 newborn types (reference T+AGA+nonLBW), by country**

Prevalence = the number of livebirths reported in each group of interest / total number of livebirths

Neonatal Mortality NMR (NMR): the number of persons who experienced the event (neonatal death) divided by the total number of persons exposed to the risk of that event per 1000

Population attributable risk (PAR): was calculated for each level of exposure with the following formula where *pr* is the prevalence and *RR* is the relative risk ^8^

$PAR for type of interest= \frac{pr\left( type of interest \right)\left( RR\left( type of interest-1 \right) \right)}{\sum_{all types} pr\left( type \right)x RR(type)}$

Relative risk: the absolute risk within a specific category divided by the absolute risk in the reference group, expressed as a ratio

| **Country** | **Total** | | **T+AGA+nonLBW** | | | |
| --- | --- | --- | --- | --- | --- | --- |
|  | **Livebirths** | **Deaths** | **Livebirths** | **Deaths** | **Prevalence** | **Rate** |
|  | **(n)** | **(n)** | **(n)** | **(n)** | **(n)** | **per 1000** |
| Australia | 1,216,172 | 2,096 | 829,536 | 280 | 68.2 | 0.3 |
| Brazil | 21,345,439 | 148,418 | 14,582,011 | 26,458 | 68.3 | 1.8 |
| Canada | 4,388,387 | 10,000 | 2,951,138 | 696 | 67.2 | 0.2 |
| Czech Republic | 109,492 | 168 | 80,129 | 32 | 73.2 | 0.4 |
| Denmark | 1,100,854 | 2,598 | 706,526 | 434 | 64.2 | 0.6 |
| England and Wales | 3,212,492 | 7,169 | 2,230,635 | 1,301 | 69.4 | 0.6 |
| Estonia | 82,298 | 95 | 52,059 | 20 | 63.3 | 0.4 |
| Lebanon | 25,515 | 114 | 18,158 | 25 | 71.2 | 1.4 |
| Mexico | 5,560,916 | 34,036 | 4,306,929 | 21,561 | 77.4 | 5.0 |
| Netherlands | 1,846,558 | 5,483 | 1,227,273 | 799 | 66.5 | 0.7 |
| Qatar | 94,337 | 297 | 66,955 | 37 | 71.0 | 0.6 |
| Scotland | 1,124,817 | 2,599 | 753,001 | 490 | 66.9 | 0.7 |
| Sweden | 2,102,671 | 2,755 | 1,410,281 | 663 | 67.1 | 0.5 |
| Uruguay | 493,040 | 664 | 338,993 | 107 | 68.8 | 0.3 |
| USA | 80,225,526 | 252,745 | 53,971,259 | 32,604 | 67.3 | 0.6 |

| **Country** | **PT+SGA+LBW** | | | | | | | |
| --- | --- | --- | --- | --- | --- | --- | --- | --- |
|  | **Livebirths** | **Deaths** | **Prevalence** | **Rate** | **PAR** | **Relative risk** | **95%CI** | |
|  | **(n)** | **(n)** | **(n)** | **per 1000** | **(%)** | **(n)** | **(n)** | |
| Australia | 7,747 | 186 | 0.6 | 24.0 | 8.7 | 69.5 | 57.8 | 83.5 |
| Brazil | 184,780 | 23,092 | 0.9 | 125.0 | 14.4 | 61.3 | 60.3 | 62.4 |
| Canada | 26,860 | 766 | 0.6 | 28.5 | 7.7 | 117.6 | 106.2 | 130.2 |
| Czech Republic | 730 | 20 | 0.7 | 27.4 | 11.6 | 66.8 | 38.4 | 116.3 |
| Denmark | 5,422 | 285 | 0.5 | 52.6 | 10.6 | 81.3 | 70.2 | 94.2 |
| England and Wales | 27,402 | 662 | 0.9 | 24.2 | 8.9 | 38.4 | 34.1 | 43.3 |
| Estonia | 286 | 2 | 0.3 | 7.0 | 2.1 | 18.1 | 4.2 | 77.0 |
| Lebanon | 271 | 14 | 1.1 | 51.7 | 11.8 | 35.7 | 18.8 | 68.0 |
| Mexico | 37,018 | 790 | 0.7 | 21.3 | 1.7 | 4.2 | 3.9 | 4.5 |
| Netherlands | 10,899 | 528 | 0.6 | 48.4 | 9.4 | 71.0 | 63.7 | 79.1 |
| Qatar | 863 | 43 | 0.9 | 49.8 | 14.0 | 85.9 | 55.6 | 132.7 |
| Scotland | 7,786 | 273 | 0.7 | 35.1 | 10.2 | 52.1 | 45.0 | 60.3 |
| Sweden | 10,807 | 346 | 0.5 | 32.0 | 12.2 | 66.0 | 58.1 | 75.1 |
| Uruguay | 3,566 | 83 | 0.7 | 23.3 | 12.2 | 72.1 | 54.2 | 95.8 |
| USA | 584,823 | 34,019 | 0.7 | 58.2 | 13.0 | 91.1 | 89.7 | 92.4 |

| **Country** | **PT+LGA+LBW** | | | | | | |
| --- | --- | --- | --- | --- | --- | --- | --- |
|  | **Livebirths** | **Deaths** | **Prevalence** | **Rate** | **PAR** | **Relative risk** | **95%CI** |
|  | **(n)** | **(n)** | **(n)** | **per 1000** | **(%)** | **(n)** | **(n)** |
| Australia | 2,094 | 147 | 0.2 | 70.2 | 6.6 | 194.4 | (160, 236.3) |
| Brazil | 71,302 | 8,992 | 0.3 | 126.1 | 5.6 | 61.8 | (60.4, 63.2) |
| Canada | 8,958 | 526 | 0.2 | 58.7 | 5.1 | 235.2 | (210.4, 262.9) |
| Czech Republic | 143 | 6 | 0.1 | 42.0 | 3.5 | 100.9 | (42.8, 237.7) |
| Denmark | 2,063 | 157 | 0.2 | 76.1 | 5.7 | 115.2 | (96.4, 137.6) |
| England and Wales | 4,570 | 344 | 0.1 | 75.3 | 4.5 | 114.0 | (99.2, 130.1) |
| Estonia | 160 | 11 | 0.2 | 68.8 | 11.2 | 167.5 | (81.5, 344.2) |
| Lebanon | 35 | 6 | 0.1 | 171.4 | 4.6 | 106.4 | (46.1, 245.7) |
| Mexico | 2,911 | 197 | 0.1 | 67.7 | 0.5 | 12.7 | (11.1, 14.5) |
| Netherlands | 3,297 | 316 | 0.2 | 95.8 | 5.4 | 134.4 | (118.5, 152.5) |
| Qatar | 268 | 13 | 0.3 | 48.5 | 4.2 | 83.8 | (45.0, 155.8) |
| Scotland | 2,159 | 157 | 0.2 | 72.7 | 5.7 | 104.2 | (87.5, 124.1) |
| Sweden | 1,426 | 104 | 0.1 | 72.9 | 3.5 | 144.7 | (118.4, 176.7) |
| Uruguay | 991 | 35 | 0.2 | 35.3 | 5.1 | 108.1 | (74.2, 157.5) |
| USA | 217,635 | 16,133 | 0.3 | 74.1 | 6.1 | 114.3 | (112.2, 116.4) |

| **Country** | **PT+AGA+LBW** | | | | | | | |
| --- | --- | --- | --- | --- | --- | --- | --- | --- |
|  | **Livebirths** | **Deaths** | **Prevalence** | **Rate** | **PAR** | **Relative risk** | **95%CI** | |
|  | **(n)** | **(n)** | **(n)** | **per 1000** | **(%)** | **(n)** | **(n)** | |
| Australia | 45,695 | 1,273 | 3.8 | 27.9 | 59.3 | 80.3 | 70.6 | 91.4 |
| Brazil | 812,417 | 61,983 | 3.8 | 76.3 | 40.0 | 39.1 | 38.6 | 39.7 |
| Canada | 162,693 | 7,108 | 3.7 | 43.7 | 70.2 | 177.5 | 164.3 | 191.9 |
| Czech Republic | 3,883 | 88 | 3.5 | 22.7 | 51.2 | 55.5 | 37.1 | 83.1 |
| Denmark | 33,773 | 1,315 | 3.1 | 38.9 | 49.4 | 61.0 | 54.8 | 68.0 |
| England and Wales | 120,084 | 3,796 | 3.7 | 31.6 | 50.9 | 49.9 | 45.2 | 55.1 |
| Estonia | 2,113 | 45 | 2.6 | 21.3 | 47.5 | 54.3 | 32.1 | 91.8 |
| Lebanon | 972 | 51 | 3.8 | 52.5 | 42.9 | 36.3 | 22.6 | 58.3 |
| Mexico | 199,076 | 5,559 | 3.6 | 27.9 | 13.1 | 5.5 | 5.3 | 5.6 |
| Netherlands | 60,803 | 3,125 | 3.3 | 51.4 | 55.6 | 75.1 | 69.5 | 81.2 |
| Qatar | 4,537 | 156 | 4.8 | 34.4 | 51.3 | 60.2 | 42.1 | 86.0 |
| Scotland | 42,253 | 1,301 | 3.8 | 30.8 | 48.6 | 45.9 | 41.4 | 50.9 |
| Sweden | 50,779 | 1,030 | 2.4 | 20.3 | 36.3 | 42.3 | 38.4 | 46.6 |
| Uruguay | 21,498 | 365 | 4.4 | 17.0 | 53.8 | 52.9 | 42.7 | 65.6 |
| USA | 3,662,791 | 134,560 | 4.6 | 36.7 | 52.1 | 58.7 | 58.0 | 59.4 |

| **Country** | **PT+AGA+nonLBW** | | | | | | | |
| --- | --- | --- | --- | --- | --- | --- | --- | --- |
|  | **Livebirths** | **Deaths** | **Prevalence** | **Rate** | **PAR** | **Relative risk** | **95%CI** | |
|  | **(n)** | **(n)** | **(n)** | **per 1000** | **(%)** | **(n)** | **(n)** | |
| Australia | 32,065 | 31 | 2.6 | 1.0 | 0.9 | 2.7 | 1.8 | 3.9 |
| Brazil | 678,612 | 4,053 | 3.2 | 6.0 | 2.0 | 3.3 | 3.2 | 3.4 |
| Canada | 118,037 | 150 | 2.7 | 1.3 | 1.3 | 5.4 | 4.5 | 6.4 |
| Czech Republic | 2,294 | 5 | 2.1 | 2.2 | 2.5 | 5.4 | 2.1 | 14.0 |
| Denmark | 23,280 | 67 | 2.1 | 2.9 | 2.1 | 4.7 | 3.6 | 6.0 |
| England and Wales | 71,058 | 191 | 2.2 | 2.7 | 2.1 | 4.4 | 3.7 | 5.2 |
| Estonia | 1,661 | 1 | 2.0 | 0.6 | 0.4 | 1.6 | 0.2 | 11.7 |
| Lebanon | 853 | 5 | 3.3 | 5.9 | 3.5 | 4.2 | 1.6 | 11.0 |
| Mexico | 120,513 | 1,027 | 2.2 | 8.5 | 1.2 | 1.7 | 1.6 | 1.8 |
| Netherlands | 42,155 | 147 | 2.3 | 3.5 | 2.3 | 5.3 | 4.5 | 6.4 |
| Qatar | 2,753 | 10 | 2.9 | 3.6 | 2.9 | 6.6 | 3.3 | 13.2 |
| Scotland | 25,649 | 62 | 2.3 | 2.4 | 1.8 | 3.7 | 2.8 | 4.8 |
| Sweden | 47,161 | 125 | 2.2 | 2.7 | 3.8 | 5.6 | 4.6 | 6.8 |
| Uruguay | 14,881 | 16 | 3.0 | 1.1 | 1.7 | 3.4 | 2.0 | 5.8 |
| USA | 2,502,528 | 5,987 | 3.1 | 2.4 | 1.8 | 4.0 | 3.8 | 4.1 |

| **Country** | **PT+LGA+nonLBW** | | | | | | | |
| --- | --- | --- | --- | --- | --- | --- | --- | --- |
|  | **Livebirths** | **Deaths** | **Prevalence** | **Rate** | **PAR** | **Relative risk** | **95%CI** | |
|  | **(n)** | **(n)** | **(n)** | **per 1000** | **(%)** | **(n)** | **(n)** | |
| Australia | 10,121 | 54 | 0.8 | 5.3 | 2.4 | 15.7 | 11.8 | 21.0 |
| Brazil | 720,230 | 4,202 | 3.4 | 5.8 | 2.0 | 3.2 | 3.1 | 3.3 |
| Canada | 40,564 | 169 | 0.9 | 4.2 | 1.6 | 17.6 | 14.9 | 20.8 |
| Czech Republic | 477 | 2 | 0.4 | 4.2 | 1.1 | 10.5 | 2.5 | 43.5 |
| Denmark | 9,037 | 74 | 0.8 | 8.2 | 2.7 | 13.2 | 10.3 | 16.9 |
| England and Wales | 20,325 | 143 | 0.6 | 7.0 | 1.8 | 11.4 | 9.4 | 13.7 |
| Estonia | 531 | 2 | 0.6 | 3.8 | 2.0 | 9.8 | 2.3 | 41.7 |
| Lebanon | 414 | 4 | 1.6 | 9.7 | 3.1 | 7.0 | 2.4 | 19.9 |
| Mexico | 22,970 | 183 | 0.4 | 8.0 | 0.2 | 1.6 | 1.4 | 1.8 |
| Netherlands | 12,128 | 109 | 0.7 | 9.0 | 1.9 | 13.7 | 11.2 | 16.7 |
| Qatar | 1,037 | 4 | 1.1 | 3.9 | 1.2 | 7.0 | 2.5 | 19.5 |
| Scotland | 7,927 | 53 | 0.7 | 6.7 | 1.9 | 10.2 | 7.7 | 13.5 |
| Sweden | 11,464 | 71 | 0.5 | 6.2 | 2.4 | 13.1 | 10.3 | 16.7 |
| Uruguay | 4,118 | 11 | 0.8 | 2.7 | 1.5 | 8.4 | 4.5 | 15.7 |
| USA | 1,072,195 | 5,941 | 1.3 | 5.5 | 2.1 | 9.1 | 8.9 | 9.4 |

| **Country** | **T+SGA+LBW** | | | | | | | |
| --- | --- | --- | --- | --- | --- | --- | --- | --- |
|  | **Livebirths** | **Deaths** | **Prevalence** | **Rate** | **PAR** | **Relative risk** | **95%CI** | |
|  | **(n)** | **(n)** | **(n)** | **per 1000** | **(%)** | **(n)** | **(n)** | |
| Australia | 17,277 | 34 | 1.4 | 2.0 | 2.2 | 8.7 | 6.5 | 11.8 |
| Brazil | 631,594 | 10,417 | 3.0 | 16.5 | 6.5 | 9.0 | 8.8 | 9.2 |
| Canada | 63,689 | 268 | 1.5 | 4.2 | 2.6 | 17.8 | 15.4 | 20.5 |
| Czech Republic | 2,428 | 12 | 2.2 | 4.9 | 6.6 | 12.3 | 6.4 | 23.9 |
| Denmark | 13,010 | 72 | 1.2 | 5.5 | 2.5 | 9.0 | 7.0 | 11.5 |
| England and Wales | 61,790 | 311 | 1.9 | 5.0 | 3.8 | 8.2 | 7.1 | 9.4 |
| Estonia | 641 | 7 | 0.8 | 10.9 | 7.3 | 28.1 | 11.9 | 66.3 |
| Lebanon | 762 | 4 | 3.0 | 5.2 | 2.7 | 3.8 | 1.3 | 10.9 |
| Mexico | 95,493 | 670 | 1.7 | 7.0 | 0.6 | 1.4 | 1.3 | 1.5 |
| Netherlands | 26,685 | 166 | 1.4 | 6.2 | 2.8 | 9.5 | 8.0 | 11.2 |
| Qatar | 2,402 | 22 | 2.5 | 9.2 | 7.1 | 16.4 | 9.7 | 27.8 |
| Scotland | 21,136 | 98 | 1.9 | 4.6 | 3.3 | 7.1 | 5.7 | 8.8 |
| Sweden | 21,455 | 131 | 1.0 | 6.1 | 4.4 | 12.9 | 10.7 | 15.6 |
| Uruguay | 9,596 | 18 | 1.9 | 1.9 | 2.3 | 5.9 | 3.6 | 9.8 |
| USA | 1,536,139 | 12,588 | 1.9 | 8.2 | 4.7 | 13.5 | 13.2 | 13.7 |

| **Country** | **T+SGA+nonLBW** | | | | | | | |
| --- | --- | --- | --- | --- | --- | --- | --- | --- |
|  | **Livebirths** | **Deaths** | **Prevalence** | **Rate** | **PAR** | **Relative risk** | **95%CI** | |
|  | **(n)** | **(n)** | **(n)** | **per 1000** | **(%)** | **(n)** | **(n)** | |
| Australia | 16,804 | 19 | 1.4 | 1.1 | 0.6 | 3.3 | 2.1 | 5.3 |
| Brazil | 892,851 | 4,077 | 4.2 | 4.6 | 1.7 | 2.5 | 2.4 | 2.6 |
| Canada | 78,679 | 73 | 1.8 | 0.9 | 0.6 | 3.9 | 3.1 | 5.0 |
| Czech Republic | 3,155 | 0 | 2.9 | 0.0 | -0.8 | 0.0 | 0.0 | 0.0 |
| Denmark | 19,082 | 41 | 1.7 | 2.1 | 1.2 | 3.5 | 2.5 | 4.8 |
| England and Wales | 72,198 | 115 | 2.2 | 1.6 | 1.0 | 2.6 | 2.1 | 3.2 |
| Estonia | 883 | 1 | 1.1 | 1.1 | 0.7 | 2.9 | 0.4 | 21.9 |
| Lebanon | 823 | 2 | 3.2 | 2.4 | 0.8 | 1.8 | 0.4 | 7.4 |
| Mexico | 276,072 | 1,524 | 5.0 | 5.5 | 0.4 | 1.1 | 1.0 | 1.2 |
| Netherlands | 30,192 | 71 | 1.6 | 2.4 | 1.0 | 3.6 | 2.8 | 4.6 |
| Qatar | 2,947 | 1 | 3.1 | 0.3 | -0.2 | 0.6 | 0.1 | 4.5 |
| Scotland | 29,275 | 46 | 2.6 | 1.6 | 1.1 | 2.4 | 1.8 | 3.3 |
| Sweden | 48,306 | 77 | 2.3 | 1.6 | 2.0 | 3.4 | 2.7 | 4.3 |
| Uruguay | 9,257 | 6 | 1.9 | 0.6 | 0.5 | 2.1 | 0.9 | 4.7 |
| USA | 1,722,245 | 3,107 | 2.1 | 1.8 | 0.8 | 3.0 | 2.9 | 3.1 |

| **Country** | **T+AGA+LBW** | | | | | | | |
| --- | --- | --- | --- | --- | --- | --- | --- | --- |
|  | **Livebirths** | **Deaths** | **Prevalence** | **Rate** | **PAR** | **Relative risk** | **95%CI** | |
|  | **(n)** | **(n)** | **(n)** | **per 1000** | **(%)** | **(n)** | **(n)** | |
| Australia | 7,297 | 8 | 0.6 | 1.1 | 0.3 | 3.2 | 1.6 | 6.6 |
| Brazil | 96,787 | 572 | 0.5 | 5.9 | 0.3 | 3.2 | 3.0 | 3.5 |
| Canada | 19,364 | 52 | 0.4 | 2.7 | 0.5 | 11.4 | 8.6 | 15.1 |
| Czech Republic | 522 | 0 | 0.5 | 0.0 | -0.1 | 0.0 | 0.0 | 0.0 |
| Denmark | 3,190 | 12 | 0.3 | 3.8 | 0.4 | 6.1 | 3.4 | 10.8 |
| England and Wales | 21,275 | 41 | 0.7 | 1.9 | 0.4 | 3.1 | 2.3 | 4.3 |
| Estonia | 235 | 0 | 0.3 | 0.0 | -0.1 | 0.0 | 0.0 | 0.0 |
| Lebanon | 122 | 0 | 0.5 | 0.0 | -0.2 | 0.0 | 0.0 | 0.0 |
| Mexico | 35,418 | 240 | 0.6 | 6.8 | 0.2 | 1.4 | 1.2 | 1.5 |
| Netherlands | 8,319 | 13 | 0.5 | 1.6 | 0.1 | 2.4 | 1.4 | 4.1 |
| Qatar | 634 | 5 | 0.7 | 7.9 | 1.6 | 14.2 | 5.6 | 35.9 |
| Scotland | 4,773 | 7 | 0.4 | 1.5 | 0.2 | 2.3 | 1.1 | 4.7 |
| Sweden | 2,422 | 5 | 0.1 | 2.1 | 0.1 | 4.4 | 1.8 | 10.6 |
| Uruguay | 2,550 | 2 | 0.5 | 0.8 | 0.2 | 2.5 | 0.6 | 10.1 |
| USA | 409,920 | 1,036 | 0.5 | 2.5 | 0.3 | 4.2 | 3.9 | 4.4 |

| **Country** | **T+LGA+nonLBW** | | | | | | | |
| --- | --- | --- | --- | --- | --- | --- | --- | --- |
|  | **Livebirths** | **Deaths** | **Prevalence** | **Rate** | **PAR (%)** | **Relative risk** | **95%CI** | |
|  | **(n)** | **(n)** | **(n)** | **per 1000** | **(%)** | **(n)** | **(n)** | |
| Australia | 247,536 | 64 | 20.4 | 0.3 | -0.9 | 0.8 | 0.6 | 1.0 |
| Brazil | 2,674,855 | 4,572 | 12.5 | 1.7 | -0.2 | 0.9 | 0.9 | 1.0 |
| Canada | 918,405 | 192 | 20.9 | 0.2 | -0.3 | 0.9 | 0.8 | 1.0 |
| Czech Republic | 15,731 | 3 | 14.4 | 0.2 | -2.0 | 0.5 | 0.1 | 1.6 |
| Denmark | 285,471 | 141 | 25.9 | 0.5 | -1.4 | 0.8 | 0.7 | 1.0 |
| England and Wales | 583,155 | 265 | 18.2 | 0.5 | -1.3 | 0.7 | 0.6 | 0.9 |
| Estonia | 23,729 | 6 | 18.2 | 0.3 | -2.2 | 0.7 | 0.3 | 1.6 |
| Lebanon | 3,105 | 3 | 12.2 | 1.0 | -1.2 | 0.7 | 0.2 | 2.3 |
| Mexico | 464,516 | 2,285 | 8.4 | 4.9 | -0.1 | 1.0 | 0.9 | 1.0 |
| Netherlands | 424,807 | 209 | 23.0 | 0.5 | -1.3 | 0.8 | 0.6 | 0.9 |
| Qatar | 11,941 | 6 | 12.7 | 0.5 | -0.2 | 0.9 | 0.4 | 2.2 |
| Scotland | 230,858 | 112 | 20.5 | 0.5 | -1.5 | 0.7 | 0.6 | 0.9 |
| Sweden | 498,570 | 203 | 23.7 | 0.4 | -1.2 | 0.9 | 0.7 | 1.0 |
| Uruguay | 87,590 | 21 | 17.8 | 0.2 | -1.0 | 0.8 | 0.5 | 1.2 |
| USA | 14,545,991 | 6,770 | 18.1 | 0.5 | -0.8 | 0.8 | 0.8 | 0.8 |

# **Additional references**

1.Benchimol EI, Smeeth L, Guttmann A, Harron K, Moher D, Petersen I, et al. The REporting of studies Conducted using Observational Routinely-collected health Data (RECORD) statement. PLoS Med. 2015;12(10):e1001885.

2.World Health Organization. ICD-10 : international statistical classification of diseases and related health problems : tenth revision 2004 [2nd ed:[Available from: <https://apps.who.int/iris/handle/10665/42980>].

3.World Health Organization. World Health Organization. International Classification of Diseases for Mortality and Morbidity Statistics (ICD-11 MMS) 2018 [ 11th ed:[Available from: [https://icd.who.int/browse11/l-m/en#](https://icd.who.int/browse11/l-m/en)!]

4.Villar J, Giuliani F, Fenton TR, Ohuma EO, Ismail LC, Kennedy SH. INTERGROWTH-21st very preterm size at birth reference charts. Lancet. 2016;387(10021):844-5.

5.Villar J, Cheikh Ismail L, Victora CG, Ohuma EO, Bertino E, Altman DG, et al. International standards for newborn weight, length, and head circumference by gestational age and sex: the Newborn Cross-Sectional Study of the INTERGROWTH-21st Project. Lancet. 2014;384(9946):857-68.

6.United Nations Children’s Fund. United Nations Inter-Agency Group for Child Mortality Estimation (UN IGME) 2022 [Available from: <https://childmortality.org/>].

7.Ashorn P, Black RE, Lawn JE, Ashorn U, Klein N, Hofmeyr J, et al. The Lancet Small Vulnerable Newborn Series: science for a healthy start. The Lancet. 2020;396(10253):743-5.

8.Laaksonen M. Population Attributable Fraction (PAF) in Epidemiologic Follow-up Studies In: Welfare NIfHa, editor. Review of th eliterature 2010.
